# Medium‐Entropy Engineering Enhances Na⁺/Electron Transport in Na_3_Fe_0.1_Mn_0.2_Co_0.2_Ni_0.3_V_1.2_(PO_4_)_2_F_3_@CNTs Cathode for Sodium‐Ion Batteries

**DOI:** 10.1002/advs.202507806

**Published:** 2025-08-30

**Authors:** Ju Yang, Najun Liu, Guanglu Jiang, Huili Peng, Kan Mi, Nana Wang, Zhongchao Bai, Xiaolei Jiang

**Affiliations:** ^1^ School of Chemistry and Chemical Engineering and Key Laboratory of Advanced Biomaterials and Nanomedicine in Universities of Shandong Linyi University Linyi 276000 China; ^2^ Centre for Clean Energy Technology School of Mathematical and Physical Sciences Faculty of Science University of Technology Sydney 2007 Australia; ^3^ Institute of Energy Materials Science University of Shanghai for Science and Technology Shanghai 200093 China

**Keywords:** cycling performance, medium entropy, NVPF, rate performance, sodium‐ion battery

## Abstract

Sodium vanadium fluorophosphate (Na_3_V_2_(PO_4_)_2_F_3_, NVPF), a promising cathode material for sodium‐ion batteries, exhibits high energy density and a stable voltage plateau, yet its practical application is hindered by intrinsic low electronic conductivity. Here, a medium‐entropy engineering strategy is introduced to address this limitation by developing a novel Na_3_Fe_0.1_Mn_0.2_Co_0.2_Ni_0.3_V_1.2_(PO_4_)_2_F_3_@CNTs (ME‐NV_1.2_PF@CNTs) composite. The medium‐entropy design synergistically optimizes structural stability and charge transport kinetics, while carbon nanotubes (CNTs) coating enhances surface conductivity. Systematic investigation reveals a parabolic relationship between electrochemical performance and entropy, with the optimal entropy value (1.2*R*) delivering a discharge capacity of 120 mAh g^−1^ at 0.1 *C* and remarkable cycling stability (60% capacity retention after 3000 cycles at 5 *C*). Structural characterization demonstrates a uniform granular morphology with surface nanopores, facilitating rapid Na^+^ diffusion. Kinetic analysis and theoretical calculations confirm that entropy‐induced lattice distortion and CNT networks synergistically accelerate Na⁺/electron transport (diffusion coefficient: ≈10^−10^ cm^2^ s^−1^) and ensure highly reversible redox reactions. In situ XRD further elucidates a dual‐phase reaction mechanism involving both single‐phase and two‐phase transitions during (de)sodiation. This work provides fundamental insights into entropy‐performance correlations and demonstrates the superiority of medium‐entropy materials in overcoming intrinsic limitations of polyanionic cathodes.

## Graphical abstract

This study proposes a medium‐entropy strategy to enhance sodium vanadium fluorophosphate cathodes. The designed Na_3_Fe_0.1_Mn_0.2_Co_0.2_Ni_0.3_V_1.2_(PO_4_)_2_F_3_@CNTs composite enhances structural stability and charge transport. Entropy‐induced distortion and carbon nanotubes (CNTs) synergistically improve Na⁺/electron kinetics. In situ XRD reveals a reversible dual‐phase reaction mechanism, demonstrating the potential of medium‐entropy engineering for advanced polyanionic cathodes.

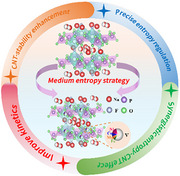

## 1 Introduction

With the escalating issues of environmental pollution and energy consumption, the effective conversion and efficient storage of energy have become critically important.^[^ ^1^, ^2^, ^3^ ^]^ Electrochemical energy storage, as a primary method of new energy storage, is currently the foremost consideration in energy storage technologies.^[^ ^4^, ^5^, ^6^ ^]^ Sodium‐ion batteries (SIBs) have emerged as a promising alternative to lithium‐ion batteries (LIBs) due to their inherent advantages of abundant sodium reserves, cost‐effectiveness, and similar electrochemical working mechanisms.^[^ ^7^, ^8^, ^9^, ^10^, ^11^ ^]^

The development of high‐performance cathode materials remains pivotal for advancing SIB technologies.^[^ ^12^, ^13^ ^]^ Among various candidates, NASICON‐type Na_3_V_2_(PO_4_)_2_F_3_ (NVPF) has attracted significant attention due to its high energy density (500 Wh kg^−1^), elevated working voltage (3.9 V vs. Na^+^/Na), and structural stability derived from robust PO_4_ tetrahedra and F^−^ ion stabilization.^[^ ^14^, ^15^ ^]^ However, its practical implementation is hindered by intrinsic limitations in electronic conductivity arising from its discontinuous V‐O‐P‐O‐V electron transfer pathways, which significantly constrain rate capability and full utilization of theoretical capacity.^[^ ^16^, ^17^ ^]^ Therefore, strategies to address this issue is crucial for improving the sodium storage capabilities of NVPF.

Extensive research has identified five primary approaches: surface coating, ion doping, morphology control, material compositing, and electrolyte optimization.^[^ ^18^, ^19^, ^20^ ^]^ Among these, ion doping has emerged as a particularly effective strategy for improving electrochemical performance. While single‐element doping can partially alleviate structural distortion, it suffers from inherent limitations.^[^ ^21^, ^22^ ^]^ These include restricted multi‐electron reactions, poor phase reversibility, and limited temperature tolerance, which collectively constrain the material's electrochemical performance.^[^ ^23^, ^24^ ^]^ Recently, the concept of configurational entropy regulation has garnered significant attention in materials science. According to the principle of configurational entropy (ΔS_config_ = ‐R∑*x_i_* ln*x_i_*), multi‐element doping can effectively elevate a material's entropy from low to high levels. This entropy enhancement mitigates cation rearrangement in crystal lattices, thereby improving phase reversibility and structural resilience under high‐voltage operation.^[^ ^25^, ^26^, ^27^ ^]^ Wu et al pioneered a high‐entropy substitution strategy that optimized the NVPF crystal structure while preserving the central V active sites. This innovation resulted in an enhancement in electronic conductivity and a reduction of Na^+^ diffusion barriers. Notably, the introduction of high‐entropy promoted disordered rearrangement of Na^+^ at Na(2) active sites, alleviating detrimental discharge behavior in low‐voltage regions while increasing the average operating voltage to 3.81 V and achieving a high energy density of 445.5 Wh kg^−1^ in Na_3_V_1.9_(Ca,Mg,Al,Cr,Mn)_0.1_(PO_4_)_2_F_3_. Nevertheless, high‐entropy phosphate materials, which typically consist of five or more transition metal elements, often suffer from lattice distortion due to disparities in element size and bond energy. Severe lattice distortion can destabilize the crystal structure, potentially leading to lattice collapse, which makes the controlled synthesis of such high‐entropy materials challenging.

In addressing these issues, Wang's group first designed a medium‐entropy compound, Na_3_Mn_2/3_V_2/3_Ti_2/3_(PO_4_)_3_/C@CNTs (ME‐NMVTP), which achieved a continuous redox reaction corresponding to 2.7 electron transfers per molecule.^[^ ^26^ ^]^ By leveraging the medium‐entropy effect, ME‐NMVTP demonstrated remarkable reversible structural evolution during sodium storage, significantly outperforming traditional low‐entropy cathode materials. However, research on medium‐entropy polyanionic compounds remains in its infancy, particularly with no systematic investigations reported on medium‐entropy effects within the NVPF system. Fundamental scientific questions persist regarding: 1) the unclear regulation mechanisms of configurational entropy on charge transport; 2) the undefined quantitative correlations between entropy variation and interfacial electric double‐layer reconstruction (affecting charge transfer resistance) or lattice kinetic parameters (governing ion diffusion barriers).

To address these challenges, we pioneered an entropy‐driven design strategy to precisely regulate the configurational entropy of NVPF through systematic multi‐cation substitution at the V site. Leveraging a solvothermal synthesis approach coupled with high‐temperature calcination, we successfully developed a novel series of medium‐entropy fluorophosphate compounds, Na_3_(VFeMnCoNi)_2_(PO_4_)_2_F_3_, representing a significant advancement in the field of medium‐entropy polyanionic cathode materials. Our comprehensive experimental and theoretical analyses demonstrate that entropy modulation plays a pivotal role in enhancing the electrochemical performance of NVPF, with optimal performance achieved at an entropy value of ≈1.2*R*, exhibiting a distinct parabolic relationship across the medium‐entropy range (1.0–1.5*R*). To further improve cycling stability, a carbon nanotube coating technique was introduced, which effectively enhanced the material stability while promoting electron transport and sodium ion diffusion rates. More importantly, our findings reveal that increased configurational entropy not only effectively mitigates structural stress during phase transitions through enhanced lattice flexibility but also optimizes the distribution of electrochemical active sites via multi‐element synergistic effects. This dual mechanism represents a fundamental breakthrough in understanding the structure‐property relationships in medium‐entropy cathode materials. The successful synthesis and characterization of these medium‐entropy fluorophosphates establishes a new paradigm for the design of high‐performance energy storage materials, bridging the critical gap between conventional low‐entropy and high‐entropy systems.

## 2 Results and Discussion

**Figure 1:**
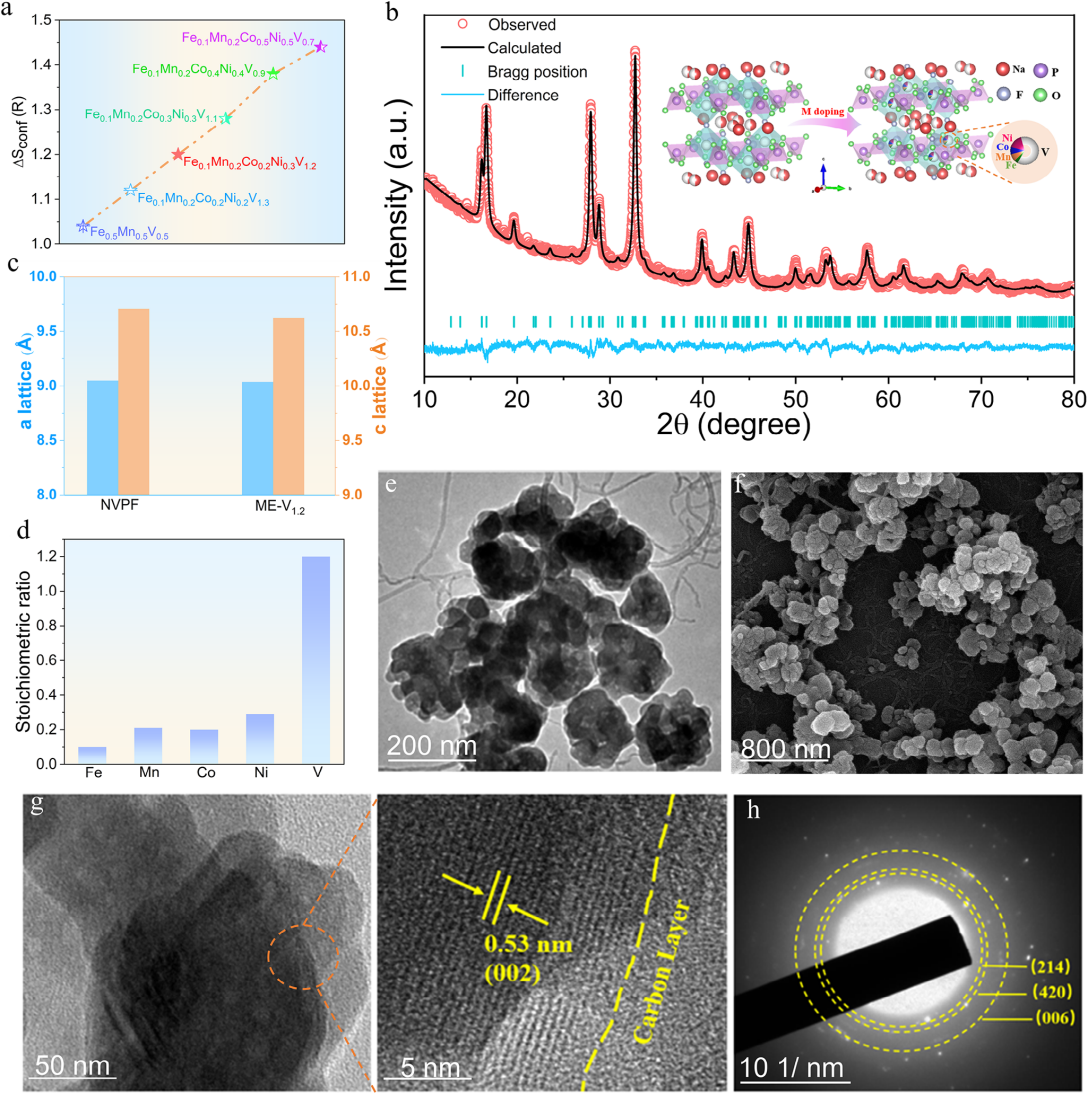
a) The entropy comparison diagram of the sample material; b) XRD refinement spectra of ME‐NV_1.2_PF@CNTs, inset figure is the crystal structure parameters of NVPF and ME‐NV_1.2_PF@CNTs; c) the crystal structure simulation diagram of ME‐NV_1.2_PF@CNTs; d) the elemental analysis results of the ME‐NV_1.2_PF@CNTs; e) the TEM of ME‐NV_1.2_PF@CNTs, f) SEM of ME‐NV_1.2_PF@CNTs, g) HRTEM of ME‐NV_1.2_PF@CNTs, h) SAED of ME‐NV_1.2_PF@CNTs.

**Figure** **1**a presents the entropy variation curves of the synthesized medium‐entropy samples, illustrating the systematic modulation of configurational entropy achieved through multi‐metal doping. The XRD patterns of the samples, shown in Figures S1 and S2 (Supporting Information), reveal that all synthesized medium‐entropy samples retain a crystal structure consistent with that of pristine NVPF. The characteristic diffraction peaks exhibit excellent alignment with the reference pattern, and no significant impurity peaks are observed, confirming the structural integrity of the doped materials. These results indicate that the entropy regulation strategy, involving the substitution of multiple metal elements at the V sites, successfully yields medium‐entropy electrode materials without disrupting the base NVPF structure. Figure S1 (Supporting Information) also reveals systematic peak broadening (increased FWHM) with decreasing vanadium content, which is attributed to local structural distortions rather than a complete loss of crystallinity. This is supported by the refined lattice parameters (Table S1, Supporting Information), which show measurable variations, suggesting that configurational entropy does influence the crystal structure. The space group of the material remains P_42_/mnm, validating the scientific rigor and rational design of the synthesis process. Notably, the carbon nanotubes (CNTs) coating significantly sharpens the XRD characteristic diffraction peaks, indicating enhanced crystallinity. This structural improvement provides a robust foundation for the subsequent optimization of electrochemical performance.

To further elucidate the structural modifications induced by medium‐entropy effect, Rietveld refinement of the XRD data was performed on the ME‐NV_1.2_PF@CNTs sample, as shown in Figure 1b. The refinement yielded excellent fitting parameters (R_wp_ = 2.91%, R_p_ = 2.34%) confirming the reliability of the structural analysis. Detailed crystallographic data, including atomic coordinates and site occupancies for ME‐NV_1.2_PF@CNTs and pristine NVPF, are provided in Tables S1 and S2 (Supporting Information). The refined lattice parameters for ME‐NV_1.2_PF@CNTs are a = b = 9.036 Å and c = 10.625 Å, showing a slight contraction compared to the standard NVPF sample (a = b = 9.05 Å, c = 10.71 Å). Inset of Figure 1b contrasts the crystal structures of the standard NVPF sample and the ME‐NV_1.2_PF@CNTs sample. The NVPF framework consists of [PO_4_] tetrahedra and [VO_6_] octahedra interconnected through corner or edge sharing, forming an open 3D network that facilitates efficient sodium ion diffusion. In the ME‐NV_1.2_PF@CNTs material, multiple metal elements partially substitute the V sites without disrupting the overall structure, introducing active sites for multi‐electron reactions and enhancing electrochemical performance. Figure 1c presents a comparative bar chart of the lattice parameters, illustrating the reduced unit cell volume of ME‐NV_1.2_PF@CNTs (867.51 Å^3^) compared to pristine NVPF (876.18 Å^3^). This lattice contraction is a direct consequence of medium‐entropy engineering, which introduces subtle yet effective structural modifications while preserving the material's fundamental framework. The engineering strategy significantly enhances the Na2 site occupancy ratio, increasing it from 0.50 to 0.59. This optimization facilitates more efficient Na⁺ extraction and insertion processes in ME‐NV_1.2_PF@CNTs, thereby improving its electrochemical performance over pristine NVPF. To validate the XRD refinement results, ICP quantitative analysis was conducted on each medium‐entropy sample. The results, presented in Table S3 (Supporting Information) and Figure 1d, demonstrate excellent agreement between the actual and theoretical proportions of each metal element. This confirms precise control over the doping process, achieving quantitative substitution of V sites with the intended metal elements.

Figure S3a–f (Supporting Information) displays the transmission electron microscopy (TEM) images of the synthesized NVPF base sample and medium‐entropy samples with varying entropy values. The NVPF base sample exhibits a self‐assembled petal‐like morphology. As the configurational entropy is regulated through multi‐metal element doping, the morphology evolves into irregular spherical or sheet‐like assemblies, accompanied by the formation of fine surface pores. This structural evolution is attributed to the entropy‐driven redistribution of metal elements during the synthesis process, which influences the nucleation and growth kinetics of the particles. Additionally, the fine surface pores serve as efficient channels for sodium ion diffusion, further contributing to improved kinetics. Figure S4a (Supporting Information) and Figure 1e,f present the TEM and SEM images of the ME‐NV_1.2_PF and ME‐NV_1.2_PF@CNTs samples. The ME‐NV_1.2_PF sample exhibits an irregular granular morphology, while the incorporation of CNTs results in a composite structure where the CNTs uniformly encapsulate the particle surfaces without altering their intrinsic shape. This CNT coating not only preserves the structural integrity of the particles but also provides a flexible conductive network that mitigates mechanical stress induced by ion deintercalation during charge and discharge processes. As shown in Figure S4b (Supporting Information), dynamic light scattering (DLS) analysis reveals a narrow particle size distribution of the ME‐NV_1.2_PF @CNTs composite, with an average diameter of 95.22 nm. This optimal nanoscale confinement is achieved through the synergistic combination of entropy‐driven regulation and carbon nanotube encapsulation. This significant reduction in particle dimensions is anticipated to substantially decrease the sodium‐ion diffusion pathways, consequently leading to enhanced electrochemical performance metrics, particularly in terms of ionic conductivity and charge transfer efficiency. Figure 1g shows the high‐resolution TEM image of the ME‐NV_1.2_PF@CNTs sample, where the lattice fringe spacing corresponds to the (002) crystal plane of the NVPF material. This observation confirms that the entropy regulation strategy preserves the crystal structure of the base material. The selected area electron diffraction (SAED) pattern of the ME‐NV_1.2_PF@CNTs sample, shown in Figure 1h, further validates the structural integrity by revealing the presence of the (214), (420), and (006) crystal planes of NVPF. Figure S5 (Supporting Information) shows the energy‐dispersive X‐ray spectroscopy (EDS) mapping of the ME‐ NV_1.2_PF@CNTs sample, demonstrating the uniform distribution of elements such as V, Fe, Co, Ni, Mn, C, O, P, Na, and F. This uniform elemental distribution confirms the successful incorporation of multiple metal elements into the NVPF structure without phase segregation.

To further characterize the porosity and surface properties of the material, Brunauer–Emmett–Teller (BET) analysis was performed on the ME‐NV_1.2_PF@CNTs sample, as shown in Figure S6 (Supporting Information). The results reveal a specific surface area of 46.9231 m^2^ g^−1^ and an average pore size of 15.455 nm, indicating the presence of the moderate but measurable mesopores. These mesopores facilitate the inward wrapping of CNTs, forming a robust encapsulation structure that enhances ion transport and electron conduction. To quantify the carbon content and its structural characteristics, thermogravimetric analysis (TGA, Figure S7, Supporting Information) and Raman spectroscopy (Figure S8, Supporting Information) were conducted. The TGA result indicates a carbon content of ≈9.5% in the ME‐ NV_1.2_PF@CNTs sample. The Raman spectrum exhibits two prominent peaks at 1355 cm^−1^ (D band) and 1587 cm^−1^ (G band), corresponding to sp^3^ hybridized carbon (indicative of defects and disorder) and sp^2^ hybridized carbon (indicative of graphitic crystallinity), respectively.^[^ ^28^, ^29^ ^]^ The intensity ratio of the D band to the G band (I_D_/I_G_) is 1.08, suggesting a balanced degree of structural disorder and crystallinity in the carbon material. This balance is beneficial for optimizing electrochemical performance, as it enhances conductivity while maintaining sufficient defect sites for ion storage and diffusion.

**Figure 2:**
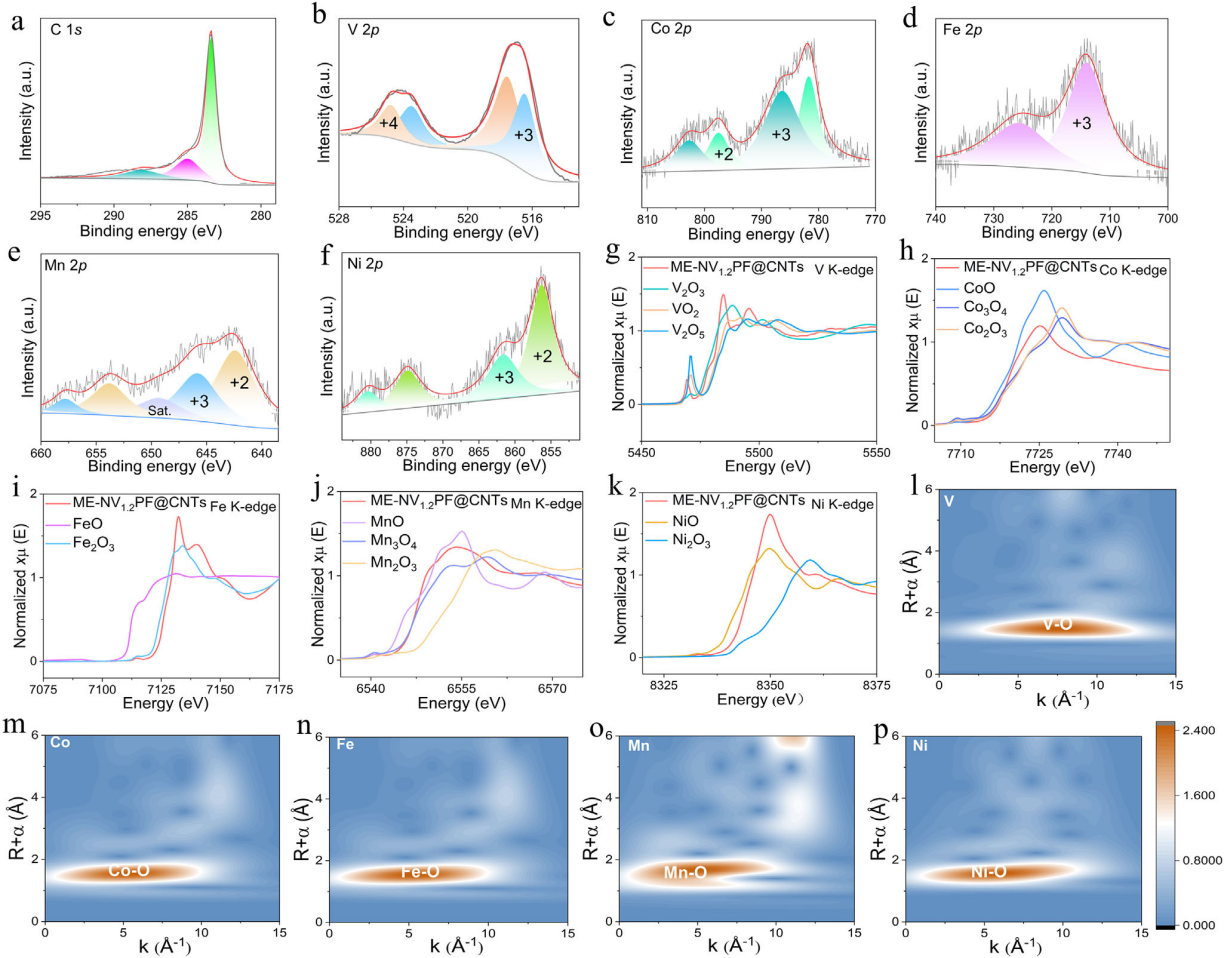
The XPS high‐resolution spectra of ME‐NV_1.2_PF@CNTs: a) C1s, b) V2p, c) Co2p, d) Fe2p, e) Mn2p and f) Ni2p. K‐edge XANES spectra of ME‐NV_1.2_PF@CNTs sample for g) V, h) Co, i) Fe, j) Mn, and k) Ni elements. The WT‐EXAFS spectra of ME‐NV_1.2_PF@CNTs sample for l) V, m) Co, n) Fe, o) Mn, and p) Ni elements.

To systematically investigate the specific valence states of the doped elements in the ME‐NV_1.2_PF@CNTs sample, a comprehensive characterization was performed using X‐ray photoelectron spectroscopy (XPS) and X‐ray absorption spectroscopy (XAS). The XPS analysis was conducted on the ME‐NV_1.2_PF@CNTs sample, with detailed results presented in Figure S9 (Supporting Information) and **Figure** **2**. Figure S9 (Supporting Information) shows the XPS survey spectrum, confirming the presence of elements such as Na, O, V, C, and P. For elements not clearly observed in the survey spectrum, high‐resolution XPS analysis was employed to confirm their oxidation states. Figure 2a displays the high‐resolution C1*s* spectrum, which was deconvoluted into three characteristic peaks at binding energies of 288.18, 284.98, and 283.48 eV, corresponding to O─C═O, C─C, and C═C chemical bonds, respectively.^[^ ^30^, ^31^ ^]^ In Figure 2b, the V2*p_3/2_* peak is divided into two distinct components at 516.48 and 517.58 eV, while the V2*p_1/2_* peak exhibits two components at 523.48 and 524.78 eV, corresponding to V^4+^ and V^3^⁺, respectively.^[^ ^26^, ^32^ ^]^ The medium entropy effect activates V^4+^ ions in V positions, which is beneficial for the redox reaction of V^5+^/V^4+^ under high voltage. Figure 2c displays the Co2*p* spectrum, where the Co2*p_1/2_* peak is deconvoluted into components at 802.78 and 797.48 eV, and the Co2*p_3/2_* peak shows components at 786.48 and 781.68 eV, corresponding to Co^3+^ and Co^2+^, respectively.^[^ ^33^ ^]^ This suggests that Co exists in a mixed valence state of +3/+2 in the electrode sample. The high‐resolution Fe2*p* spectrum, shown in Figure 2d, exhibits a relatively low signal‐to‐noise ratio, indicating a low Fe content in the sample. However, the split peaks at 725.18 eV (Fe2*p_1/2_*) and 713.98 eV (Fe2*p_3/2_*) are identified, confirming the presence of Fe in the +3 oxidation state.^[^ ^34^ ^]^ The Mn2*p* spectrum in Figure 2e presents split peaks at 653.38 eV (Mn2*p_1/2_*) and 642.48 eV (Mn2*p_3/2_*), with a strong satellite peak.^[^ ^35^, ^36^ ^]^ Comparison with relevant Mn valence state energy data confirms that Mn exists as +2 valence in the electrode sample. Figure 2f shows the Ni2*p* spectrum, where the Ni2*p_3/2_* is divided into two peaks at 861.78 and 856.38 eV, while the Ni2*p_1/2_* peak is resolved into components at 880.28 and 874.58 eV, corresponding to Ni^3+^ and Ni^2+^, respectively.^[^ ^37^, ^38^ ^]^

**Figure 3:**
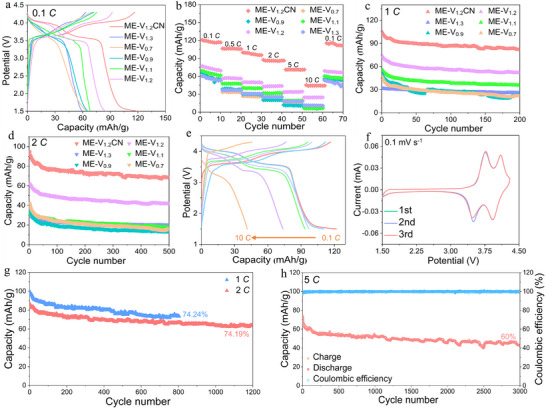
a) The charge–discharge curves at 0.1 *C*, b) rate performance, c) the cycle performance diagram at 1 *C*, d) cyclic performance at 2 *C*, e) the charge–discharge curves at 0.1 *C*, 0.5 *C*, 1 *C*, 2 *C*, 5 *C* and 10 *C* of ME‐NV_1.2_PF@CNTs; f) the first three CV curves at 0.1 mV s^−1^ of ME‐V_1.2_PF@CNTs, g) the long cycle performance at 1 *C* and 2 *C* of ME‐NV_1.2_PF@CNTs and h) Long cycle performance and coulombic efficiency at 5 *C* of ME‐NV_1.2_PF@CNTs.

To rigorously address the discrepancies between XPS and XANES results, we have conducted additional XANES analyses with careful energy calibration for each element to better determine the dopant valence states, as shown in Figure 2g–k. While XPS predominantly probes the surface valence state composition, XANES provides a more comprehensive assessment of the bulk valence states. Consequently, a synergistic approach integrating both techniques is essential for accurate characterization. For V species, we incorporated reference spectra of V_2_O_3_ (V^3+^) and VO_2_ (V^4+^) for comparative analysis. Through systematic examination of the absorption edge positions, we determined that ME‐NV_1.2_PF@CNTs exhibits a valence state intermediate between V^3+^ and V^4+^, suggesting a mixed valence configuration. In the case of Fe, the K‐edge absorption edge of ME‐NV_1.2_PF@CNTs is higher than that of Fe_2_O_3_ (Fe^3+^), suggesting a possible higher oxidation state (e.g., partial Fe^4+^) or local structural distortion. However, the spectrum is closer to Fe^3+^ than to FeO (Fe^2+^), supporting a dominant Fe^3+^ state with possible mixed contributions. Detailed analysis of the near‐edge absorption features for Co and Mn revealed that both elements exist in mixed valence states, namely +2 and +3 oxidation states. Minor discrepancies between the XANES spectra and XPS analysis results were observed, likely attributable to the incorporation of multiple metal elements. This doping effect reduces the local symmetry of the sample's internal structure, inducing minor structural distortions while maintaining the overall framework's integrity.^[^ ^39^, ^40^, ^41^ ^]^ The k^3^‐weighted Fourier‐transformed EXAFS (FT‐EXAFS) spectra of ME‐NV_1.2_PF@CNTs (Figure S10a–e, Supporting Information) were analyzed within the k‐range of 2–10 Å^−1^. Only the |𝜒(R)| corresponding to the first shell M─O bond at ≈1.5 Å was observed. The analysis reveals that the M─O/F bonds are situated within the same shell, with the average bond length of M─F being slightly shorter than that of M─O. The nearest neighbor atomic pair distances and corresponding Debye‐Waller factors obtained from the fitting are listed in Table S4 (Supporting Information). The average M─O/F bond length (≈2.0 Å) calculated from XRD Rietveld refinement is consistent with that in ME‐NV1.2PF@CNTs. To further explore the local coordination environment, we performed wavelet‐transformed EXAFS (WT‐EXAFS) analysis for all five metals. WT‐EXAFS, as shown in Figure 2l–p, provides detailed information about the local atomic configurations and serves as an efficient method for comparing the coordination environments of transition metals. The WT‐EXAFS analysis revealed peaks exclusively corresponding to M─O bonds, demonstrating similar coordination environments for all substituted metals to that of V in the crystal lattice. The combined XAS and XPS results indicate that the lattice contraction in the ME‐NV_1.2_PF@CNTs crystal structure arises from the smaller average ionic radii of Mn^2+^, Fe^3+^, Co^3+^, and Ni^2+^ compared to V^3+^ in the six‐coordination environment. These findings provide compelling evidence for the successful substitution of transition metal elements into the central V site in the Na_3_V_2_(PO_4_)_3_F_3_ lattice structure, offering valuable insights into the material's structural and electronic properties. **Figure** **3** presents a comparative analysis of the electrochemical performance of six medium‐entropy samples, evaluated within a voltage range of 1.5–4.3 V. Figure 3a illustrates the first‐cycle charge–discharge specific capacities of the medium‐entropy samples at a current density of 0.1 *C*. A clear trend is observed: with consistent doping elements, the first‐cycles pecific capacity of the electrode materials within the medium‐entropy range initially increases and then decreases, exhibiting a parabolic relationship with entropy. Notably, the ME‐NV_1.2_PF sample exhibits the highest first‐cycle discharge specific capacity of 85 mAh g^−1^ without CNTs coating. After coating CNTs, its discharge specific capacity significantly increases to 120 mAh g^−1^, confirming the synergistic effect of entropy regulation and conductive CNTs modification in optimizing the electrochemical performance of NVPF electrode. Figure 3b compares the rate performance of the medium‐entropy samples, revealing a consistent trend with first‐cycle charge–discharge specific capacity. The sample with an entropy value of 1.2*R* exhibiting the most outstanding performance, and its rate performance is further enhanced after CNTs coating. This improvement highlights the critical role of entropy regulation in optimizing the rate capability of electrode materials, with the CNT coating providing additional conductivity and structural stability. Figure 3c displays the cycling performance of the medium‐entropy samples at a current density of 1 *C*. After 200 cycles, the trend aligns with the rate performance results, demonstrating the robustness of the entropy‐regulated materials. To further validate these findings, Figure 3d presents the performance test results after 500 cycles at a current density of 2 *C*. The results confirm the parabolic relationship between entropy and electrochemical performance within the medium‐entropy range, with the ME‐NV_1.2_PF sample exhibiting optimal cycling stability, rate capability, and specific capacity. The incorporation of CNTs further enhances the sodium storage performance, achieving a significant improvement in electrochemical performance.

Based on systematic characterization and fundamental electrochemical performance analysis, the ME‐NV_1.2_PF sample with an entropy value of 1.2*R* was identified as the optimal cathode material for sodium‐ion batteries. The CNTs coating on its surface provides efficient modification, enhancing conductivity and structural integrity. To further explore the electrochemical performance evolution and sodium storage mechanism of this material, detailed electrochemical tests were conducted. Figure 3e shows the charge‐discharge curves of the ME‐NV_1.2_PF@CNTs electrode at different current densities. The electrode exhibits average discharge specific capacities of 120, 110, 100, 95, 80, and 43 mAh g^−1^ at current densities of 0.1 *C*, 0.5 *C*, 1 *C*, 2 *C*, 5 *C*, and 10 *C*, respectively. As the current density increases, the specific capacity gradually decreases, with a sharp decline observed at 5 *C*, likely due to kinetic limitations. Two distinct voltage plateaus are visible in the charge–discharge curves, indicating the involvement of at least two pairs of redox reactions within the material. Figure 3f presents the cyclic voltammetry (CV) curves of the ME‐NV_1.2_PF@CNTs electrode at a scan rate of 0.1 mV s^−1^ for the first three cycles. The CV profiles distinctly reveal two well‐defined redox couples, characterized by oxidation/reduction potentials at ≈3.76/3.5 V and 4.1/3.9 V, respectively. These electrochemical features are attributed to the sequential redox transitions of V^4+/3+^ and Mn^3+/2+^ in the lower potential region, followed by Mn^4+/3+^ and V^5+/4+^ redox processes at higher potentials, respectively. This suggests that entropy regulation activates multiple redox‐active ions, and their synergistic effect significantly enhances the sodium storage capacity of the electrode material. Additionally, the high overlap of the first three CV curves confirms the excellent structural stability and charge–discharge reversibility of the ME‐NV_1.2_PF@CNTs sample, primarily attributed to the robust carbon layer framework constructed by CNTs. This framework effectively buffers structural stress during charge–discharge processes, stabilizing the material structure. Figure 3g displays the long‐term cycling performance of the ME‐NV_1.2_PF@CNTs sample. After 800 cycles at a current density of 1 *C*, the capacity retention rate reaches 74.24%. After 1200 cycles at 2 *C*, the final discharge specific capacity maintains 74.19% of the initial value, demonstrating exceptional cycling stability. Figure 3h shows the long‐term cycling performance and Coulombic efficiency of the ME‐NV_1.2_PF@CNTs sample at 5 *C*. After 3000 cycles, the Coulombic efficiency remains consistently ≈100%, with a capacity retention rate of 60%. These results highlight the ME‐NV_1.2_PF@CNTs electrode as a promising candidate for sodium‐ion batteries, exhibiting not only excellent rate performance but also outstanding long‐term cycling stability.

**Figure 4:**
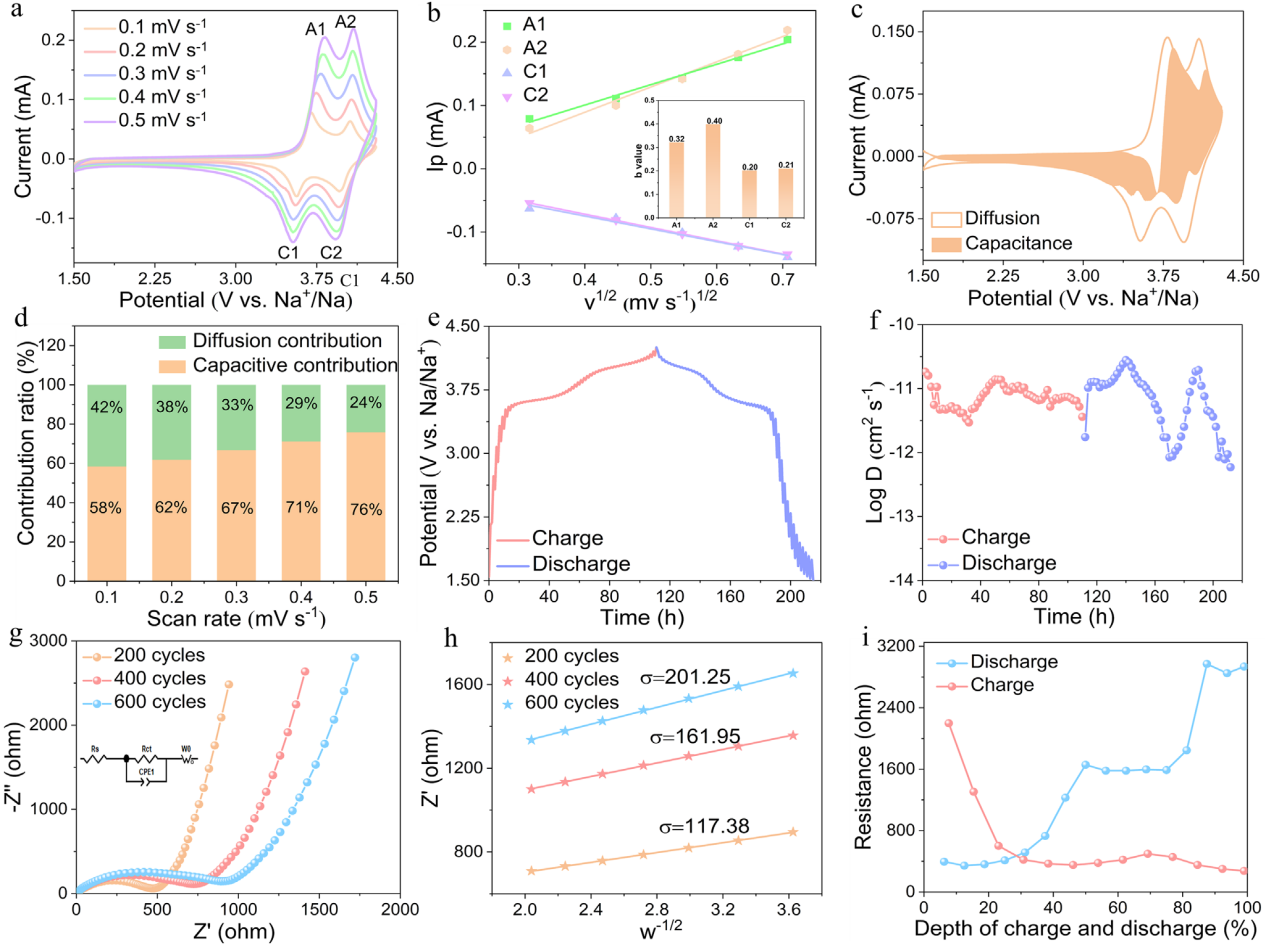
ME‐NV_1.2_PF@CNTs: a) CV test curves at different scan rates (0.1–0.5 mV s^−1^), b) the relationship curve between v^1/2^ and *i*, c) the pseudo‐capacitance contribution curve at 0.3 mV s^−1^ and d) pseudo‐capacitance contribution histogram at 0.1–0.5 mV s^−1^, e) GITT test curve and f) curve of variation in sodium ion diffusion coefficient, g) EIS test curve and h) warburg efficiency fitting curve, i) depth of charge and discharge of resistance change curve.

**Figure** **4** presents a detailed kinetic analysis of the ME‐NV_1.2_PF@CNTs sample, providing insights into its electrochemical behavior and sodium ion diffusion kinetics. Figure 4a shows the CV curves of the ME‐NV_1.2_PF@CNTs sample at scan rates ranging from 0.1 to 0.5 mV s^−1^. The CV curves remain their overall shape across different scan rates, indicating stable electrochemical behavior. However, as the scan rate increases, a slight shift in the redox peak positions of the redox peaks shift slightly is observed, suggesting the presence of partial polarization in the electrode material under high scan rate conditions. To quantify the kinetic behavior, the relationship between the peak current (*i*) and scan rate (*v*) was plotted, as shown in Figure 4b. The fitted b‐values, all below 0.5, confirm that the electrode material exhibits battery‐like behavior, dominated by diffusion‐controlled processes rather than surface capacitive effects. The specific calculation formula is as follows.^[^ ^42^ ^]^

(1) $$i=av^{b}$$

(2) $$i\left(v\right)=k_{1}v+k_{2}v^{1/2}$$

Among them, a, b, *k_1_*, and *k_2_* are constants, where *k_1_v* and *k_2_v^1/2^* represent the contributions of pseudocapacitive behavior and battery‐like diffusion behavior, respectively. The capacitive contribution of the electrode material was further evaluated. Figure 4c illustrates the pseudocapacitive contribution at a scan rate of 0.3 mV s^−1^, where the orange shaded area represents scan rates from 0.1 to 0.5 mV s^−1^, the values are 58%, 62%, 67%, 71%, and 76%, respectively. This trend highlights the enhanced surface‐controlled charge storage at faster scan rates, which is beneficial for high‐rate applications.

To explore the sodium ion diffusion kinetics, galvanostatic intermittent titration technique (GITT) and electrochemical impedance spectroscopy (EIS) tests were conducted. Figure 4e,f presents the GITT results, obtained at a current density of 0.1 *C* with a pulse duration of 10 min and a relaxation time of 60 min yielding a voltage‐time curve. The sodium ion diffusion coefficient was then calculated using the relevant formula, as follows:^[^ ^43^ ^]^

(3) $$D=\frac{4}{\pi t}\times {\left(\frac{n_{m}V_{M}}{S}\right)}^{2}{\left[\frac{\Delta Es}{\Delta Et}\right]}^{2}\left(t\ll \frac{L^{2}}{D}\right)$$

Here, *D* represents the diffusion coefficient of Na⁺ (cm^2^ s^−1^), *V_M_* is the molar volume of the electrode material (cm^3^ mol^−1^), *n_m_* denotes the number of moles of the electrode material, *S* is the effective surface area of contact between the electrode and electrolyte (cm^2^), *t* is the relaxation time (s), *ΔE_s_* is the voltage change induced by the pulse (V), *ΔE_t_* reflects the voltage change during constant current charge–discharge (V), and *L* is the thickness of the electrode (cm). By substituting the relevant data, the calculated sodium ion diffusion coefficient (D_Na+_) ranges from 10^−10.5^ to 10^−12^ cm^2^ s^−1^, indicating relatively fast sodium ion diffusion within the material. This is attributed to the synergistic effects of entropy regulation and the 3D carbon framework provided by CNTs, which facilitate ion transport and electron conduction, thereby enhancing the material's sodium storage performance. Figure 4g,h presents the EIS results of the ME‐NV_1.2_PF@CNTs sample after 200, 400, and 600 cycles, tested at room temperature within a frequency range of 10^−2^ to 10^5^ Hz. The Warburg coefficient (σ) was obtained by the linear fitting the low‐frequency region, as shown in Figure 6c. The specific calculation formula is as follows:^[^ ^44^ ^]^

(4)

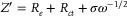

(5) $$D_{Na^{+}}=\frac{R^{2}T^{2}}{2A^{2}n^{4}F^{4}C^{2}\sigma^{2}}$$

Here, *R* represents the gas constant, *T* denotes room temperature, *A* is the area of the active cathode material (*A * = 1.13 cm^2^), *n* is the number of ions deintercalated during the charge–discharge process (*n* = 2), *F* is the Faraday constant, and *C* is the sodium ion concentration in the electrode material (*C* = 7.85 × 10^−4^ mol mL^−1^). The D_Na_^+^ was calculated for different cycle counts, as summarized in Table S5 (Supporting Information). While the internal resistance of the material increases with cycling, the overall increase is modest, contributing to the material's excellent cycling performance. To further investigate the impedance evolution during charge–discharge processes, we conducted direct current (DC) impedance tests. Figure S11 (Supporting Information) displays the DC impedance curve of the ME‐NV_1.2_PF@CNTs sample, where the horizontal axis represents the charge–discharge depth, and the vertical axis represents the voltage variation. The test was performed after 60 cycles at a current density of 0.1 *C*, recording the voltage difference before and after resting. The DC impedance at various states of charge (SOC) or depths of discharge (DOD) was calculated using the formula *R_dc_ =ΔU/ΔV*, enabling real‐time monitoring of the impedance evolution of the electrode material throughout the charge–discharge process. The results, shown in Figure 4i, indicate that the battery polarization resistance remains below 3000 Ω throughout the charge–discharge cycle, providing favorable conditions for efficient ion diffusion and minimizing energy losses. These kinetic analysis reveals that the ME‐NV_1.2_PF@CNTs sample exhibits a balanced combination of diffusion‐controlled and surface‐controlled charge storage mechanisms, with fast sodium ion diffusion and low polarization resistance.

**Figure 5:**
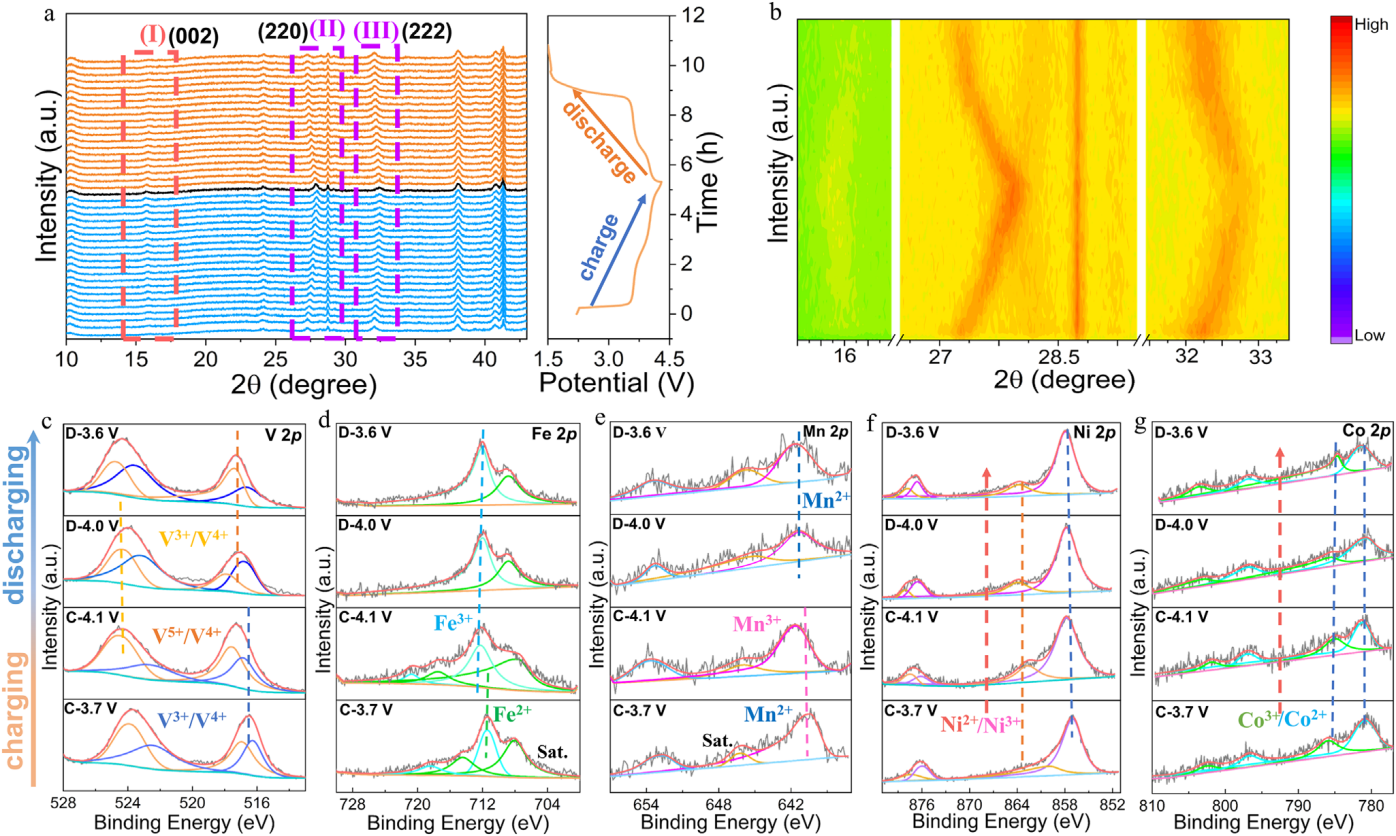
ME‐NV_1.2_PF@CNTs: a) In situ XRD test curve and b) contour map of in situ XRD test data of ME‐NV_1.2_PF@CNTs. High‐resolution XPS spectra of c) V 2p, d) Fe 2p, e) Mn 2p, f) Ni 2p, and g) Co 2p of the ME‐NV_1.2_PF@CNTs cathode collected at four states during the initial cycle.

To further monitor the crystal structure evolution of the ME‐NV_1.2_PF@CNTs sample during charge–discharge processes, in situ XRD analysis was conducted for the initial cycle. The tests were performed within a voltage range of 2–4.3 V, with a scanning angle range of 10°–44.5°, and the data collection interval was 86.4 s. **Figure** **5**a illustrates the dynamic changes in the characteristic peaks during charge–discharge, which are mainly concentrated in regions I, II, and III, corresponding to the (002), (220), and (222) crystal planes, respectively. During the charging process, the diffraction peaks of the (220) and (222) planes gradually shift toward higher angles as the voltage increases, indicating a contraction in the lattice volume due to the extraction of Na^+^ ions. Conversely, during discharge, these peaks revert to their initial positions, demonstrating the highly reversible nature of the structural changes in the ME‐NV_1.2_PF@CNTs sample. This behavior is consistent with a single‐phase reaction mechanism, as supported by the reversible lattice parameter variations throughout the cycle.^[^ ^45^, ^46^ ^]^ To elucidate the dynamic behavior of specific crystal plane, a contour map analysis of the in situ XRD data was performed, as shown in Figure 5b. In region I, specifically near the (002) plane, the appearance and subsequent disappearance of new peaks were observed, which can be attributed to a two‐phase reaction occurring in the high‐voltage region. This two‐phase reaction is recognized for its potential impact on cycling reversibility. These findings suggest that the sodium ion storage mechanism in the ME‐NV_1.2_PF@CNTs sample is governed by a dual‐reaction model: a single‐phase reaction dominates the low‐voltage region, while a two‐phase reaction becomes significant in the high‐voltage region.^[^ ^47^ ^]^

To gain further insights into the redox behavior of the transition metal ions in the ME‐NV_1.2_PF@CNTs material, ex situ XPS analysis was conducted on the metallic elements V, Fe, Co, Mn, and Ni at key states during the initial cycle. The states were selected based on four redox plateaus: i) charged to 3.7 V (C‐3.7 V), ii) charged to 4.1 V (C‐4.1 V), iii) discharged to 4.0 V (D‐4.0 V), and iv) discharged to 3.6 V (D‐3.6 V), as depicted in Figure 5c–g. Figure 5c reveals that V exists in a mixed +4/+3 valence state at C‐3.7 V. As the charging process progresses, the binding energy shifts toward higher values, indicating the oxidation of V^4+^ to V^5+^. During discharge, the binding energy gradually shifts back to lower values, suggesting a certain degree of reversibility in the overall charge reaction. At D‐4.0 V, V^5+^ is reduced back to V^4+^, and at D‐3.6 V, a mixed V^3+^/V^4+^ state is observed, confirming the complete activation of the V^3+^/V^4+^/V^5+^ redox reactions during the electrochemical process. This highlights the effectiveness of medium‐entropy engineering in enhancing the redox activity of V^4+^/V^5+^, thereby contributing to the high capacity and energy density of the material. Figure 5d shows the valence state changes of Fe, which transitions from +2 to +3 during the first redox reaction and remains stable at +3 in subsequent electrochemical reactions. According to Figure 5e, the valence state of Mn undergoes a reversible transition between +2 to +3. Figure 5f,g reveals that Co and Ni maintain a mixed +3/+2 valence state throughout the electrochemical reaction process. However, Ni gradually shifts toward higher binding energy, reflecting an increasing proportion of Ni^3+^, while Co shows minimal peak shift, suggesting its relatively stable role in the redox reactions. The activation of multiple redox‐active ion pairs, including V, Fe, Mn, Co, and Ni, provides the intrinsic conditions for the ME‐NV_1.2_PF@CNTs material to achieve superior charge–discharge specific capacity. Through a comprehensive analysis of the experimental results, the medium‐entropy strategy has been confirmed as an effective approach for enhancing the material's conductivity, ion transport properties, and structural stability.

**Figure 6:**
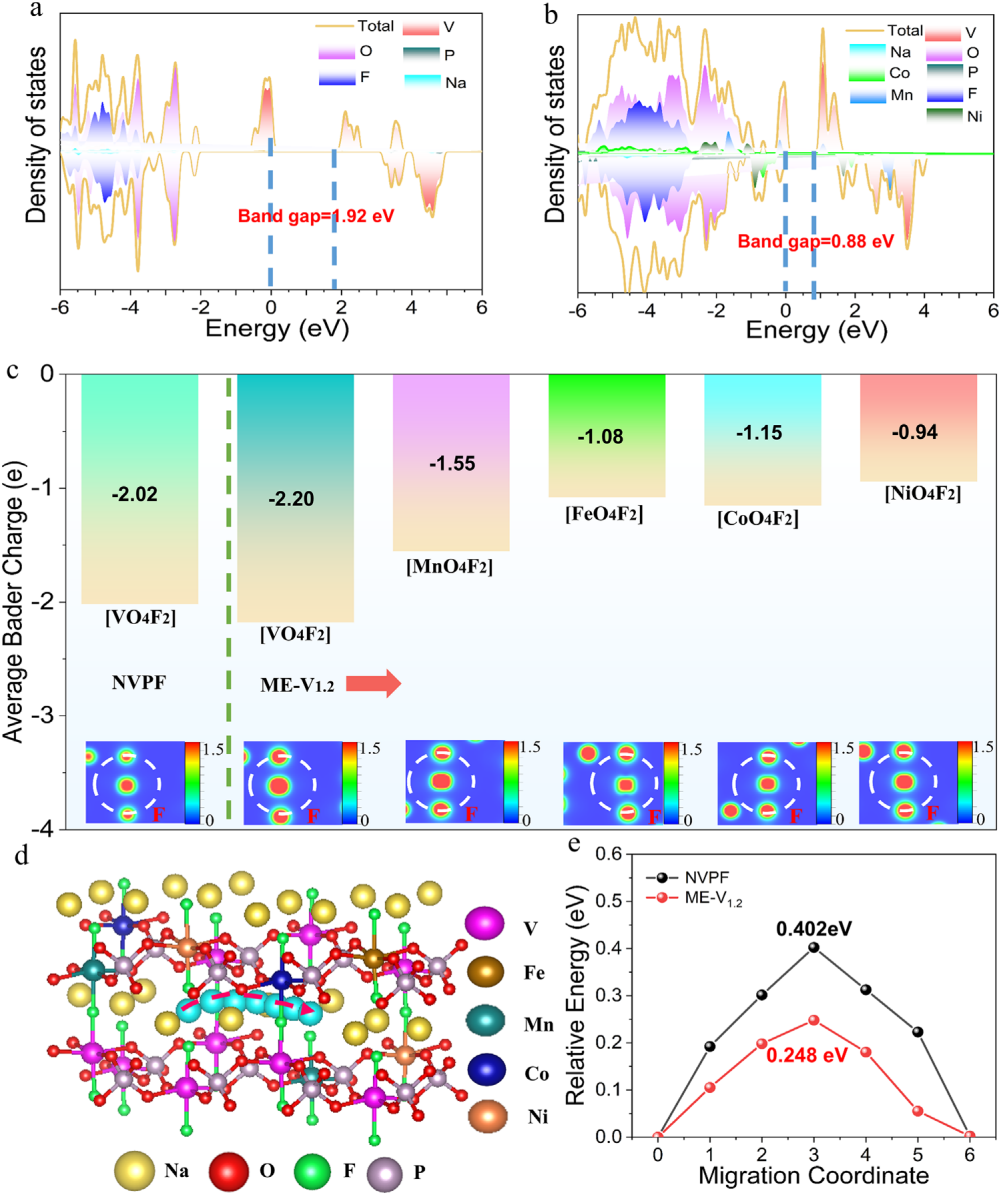
The density of states (DOS) of a) NVPF and b) ME‐NV_1.2_PF. The Bader charge analysis of the oxygen atoms in the [TMO_4_F_2_] octahedra of the (a) NVPF and (b) ME‐NV_1.2_PF crystal, the insets display the charge density contour maps on the corresponding plane for the [TMO_4_F_2_] octahedron. d) Sodium ion migration path of ME‐NV_1.2_PF, e) the Na^+^ migration energy barriers of NVPF and ME‐NV_1.2_PF.

To gain deeper insights into the intrinsic characteristics of the medium‐entropy material, density functional theory (DFT) calculations were systematically performed. Figure S12a,b (Supporting Information) presents the theoretical crystal models of the NVPF and ME‐NV_1.2_PF samples, respectively, offering a detailed visualization of their elemental compositions and structural configurations. Based on these crystal structure models, the total density of states (tDOS) for the NVPF and ME‐NV_1.2_PF samples was calculated, with the results depicted in **Figure** **6**a,b. Notably, the bandgap of the NVPF sample decreased from 1.92 to 0.88 eV, with the primary changes occurring in conduction band. This significant reduction in bandgap demonstrates that medium‐entropy regulation significantly improves the electronic conductivity of NVPF. To further elucidate the changes in electron density distribution, the charge density difference calculations and charge density distribution analyses were conducted. Figure S13 (Supporting Information) displays the differential charge density map of the ME‐NV_1.2_PF sample, with an isosurface value of 0.002 e/Å^3^, where yellow regions indicate electron accumulation and red regions denote electron depletion. Compared to the V─O bond, the Mn‐O, Fe─O, Co─O, and Ni─O bonds exhibit more pronounced yellow areas, suggesting that multi‐element doping significantly enhances the degree of electron localization. Figures 6c and S14a,b (Supporting Information) utilize Bader charge analysis to investigate the influence of the medium‐entropy effect on the bonding interactions and charge transfer within the NVPF crystal structure. In contrast to the NVPF structure, the electron density around each element in the ME‐NV_1.2_PF structure is substantially increased, further confirming that the medium‐entropy strategy significantly improves the material's electronic conductivity. Additionally, the Bader charges of the [TMO_4_F_2_] octahedral structures formed by metal dopants with F and O elements are more negative in ME‐NV_1.2_PF, ensuring greater structural stability, which is crucial for long‐term electrochemical cycling. The changes in electron density not only affect the stability of the crystal structure but also significantly influence the energy barrier for Na⁺ migration. Figure S15 (Supporting Information) and Figure 6d illustrate the Na⁺ diffusion pathways within the NVPF and ME‐NV_1.2_PF structures, respectively, revealing identical Na⁺ diffusion paths. Figure 6e compares the sodium ion migration energy barriers of these two structures. Notably, the migration energy barrier for ME‐NV_1.2_PF is only 0.248 eV, significantly lower than the 0.402 eV for NVPF along the same path. This reduced energy barrier indicates that ME‐NV_1.2_PF requires less energy during the sodium ion extraction and insertion processes, consistent with the GITT test results. The lowered migration energy barrier directly contributes to the superior kinetic behavior exhibited by the ME‐NV_1.2_PF sample. In summary, DFT analysis theoretically confirms that the medium‐entropy strategy effectively narrows the bandgap, alters electron density, and reduces the migration energy barrier within the electrode material. By carefully selecting doping elements, this strategy further enhances the material's electronic conductivity and Na⁺ diffusion capability, thereby enabling ME‐NV_1.2_PF to exhibit exceptional reaction kinetics, structural stability, and electrochemical performance.

## 3 Conclusion

In conclusion, we employed a facile solvothermal method combined with a high‐temperature calcination strategy to achieve precise entropy regulation in NVPF‐based electrode materials through controlled doping of Fe, Co, Ni, and Mn. This innovative approach enabled the systematic synthesis of a series of medium‐entropy materials. Our findings demonstrate that the entropy plays a pivotal role in determining both the microstructure and electrochemical performance of the material. Within the medium‐entropy range, as the entropy value increases, the specific capacity, rate performance, and cycling performance of the material initially improve and then decline. The ME‐NV_1.2_PF sample, with an entropy value of 1.2*R*, emerged as the optimal composition, exhibiting superior battery performance. Further modification with CNTs not only significantly enhanced the electronic conductivity but also effectively mitigated structural degradation during ion deintercalation processes. In situ XRD analysis revealed that the sodium storage process in the ME‐NV_1.2_PF@CNTs material is highly reversible, governed by a combination of single‐phase and two‐phase reaction mechanisms. Comprehensive kinetic analysis and theoretical calculations confirm that the synergistic effects of medium‐entropy regulation and CNTs modification endow the ME‐NV_1.2_PF@CNTs material with exceptional structural stability and superior Na⁺ migration kinetics. These advantageous properties translate into remarkable electrochemical performance, with the ME‐NV_1.2_PF@CNTs composite demonstrating excellent long‐term cycling stability, retaining 60% of its capacity after 3000 cycles at a high current density of 5 *C*. The successful integration of medium‐entropy regulation and CNTs modification opens new avenues for developing advanced energy storage materials with optimized electrochemical properties.

## Conflict of Interest

The authors declare no conflict of interest.

## Supporting information



Supporting Information

## Data Availability

The data that support the findings of this study are available in the supplementary material of this article.
