# Supplementary material for: Medium‐Entropy Engineering Enhances Na⁺/Electron Transport in Na3Fe0.1Mn0.2Co0.2Ni0.3V1.2(PO4)2F3@CNTs Cathode for Sodium‐Ion Batteries
Source: Adv Sci (Weinh). 2025 Aug 30;12(44):e07806. doi: 10.1002/advs.202507806 (PMC12667550; doi:10.1002/advs.202507806)
Supplement: Supplementary file 1 — Supporting Information [file ADVS-12-e07806-s001.docx]

*Supporting Information*

**Medium-entropy engineering enhances Na⁺/electron transport in** **Na_3_Fe_0.1_Mn_0.2_Co_0.2_Ni_0.3_V_1.2_(PO_4_)_2_F_3_@CNTs cathode for sodium-ion batteries**

*Ju Yang, Najun Liu, Guanglu Jiang, Huili Peng, Kan Mi, Nana Wang,^*^ Zhongchao Bai, and Xiaolei Jiang^*^*

**Experimental Section**

**Sample Preparation**

***Synthesis of Na_3_V_2_(PO_4_)_2_F_3_***

NH_4_VO_3_, Na_2_H_2_PO_4_, and NaF were added to a hydrothermal autoclave in a stoichiometric ratio of 2:2:3, using C_8_H_18_O_5_ as the solvent and lactic acid as the reducing agent. The mixture was thoroughly stirred and mixed for 0.5 h. The reaction was carried out in an oven at 180 °C for 20 h, followed by drying at 60 °C for 12 h. Finally, the sample was preheated at 200 °C for 4 h and then calcined at 400 °C for 6 h under an argon atmosphere, with a heating rate of 1 °C/min, to obtain the final product Na_3_V_2_(PO_4_)_2_F_3_ (NVPF).

***Synthesis of Na_3_(VFeMnCoNi)_2_(PO4)_2_F_3_***

A precisely measured stoichiometric mixture of 0.1404 g NH_4_VO_3_, 0.0174 g (CH_3_COO)_2_Fe, 0.034 g (CH_3_COO)_2_Mn, 0.05 g CoCl_4_·5H_2_O and 0.0746 g (CH_3_COO)_2_Ni was introduced into a round-bottom flask. Subsequently, 5 mL of lactic acid was added as the reducing agent, and the reaction mixture was maintained at 40 °C with continuous stirring for 30 min to ensure complete dissolution. Following the thorough homogenization of the precursor materials, 0.24 g Na_2_H_2_PO_4_ and 0.126 g NaF were sequentially introduced in stoichiometric proportions. The reaction system was then diluted with 35 mL of C_8_H_18_O_5_ (tetraethylene-glycol) as the solvent to achieve a homogeneous solution. The final reaction mixture was transferred into a 100 mL Teflon-lined stainless-steel autoclave and subjected to hydrothermal treatment at 180 °C for 20 h to facilitate the formation of the desired crystalline phase. After the reaction, the solution was cooled to room temperature, followed by filtration and drying. The dried sample was transferred to a corundum boat, preheated at 200 °C for 4 h with a heating rate of 1 °C/min, and then ground uniformly. Subsequently, the sample was calcined at 400 °C for 6 h to obtain the final electrode material. The chemical formulas of the samples are as follows: Na_3_Fe_0.1_Mn_0.2_Co_0.2_Ni_0.2_V_1.3_(PO_4_)_2_F_3_ (denoted as ME-NV_1.3_PF, △S=1.12R), Na_3_Fe_0.1_Mn_0.2_Co_0.2_Ni_0.3_V_1.2_(PO_4_)_2_F_3_ (denoted as ME-NV_1.2_PF, △S=1.2014R), Na_3_Fe_0.1_Mn_0.2_Co_0.3_Ni_0.3_V_1.1_(PO_4_)_2_F_3_ (denoted as ME-NV_1.1_PF, △S=1.278R), Na_3_Fe_0.1_Mn_0.2_Co_0.4_Ni_0.4_V_0.9_(PO_4_)_2_F_3_ (denoted as ME-NV_0.9_PF, △S=1.3831R), Na_3_Fe_0.1_Mn_0.2_Co_0.5_Ni_0.5_V_0.7_(PO_4_)_2_F_3_ (denoted as ME-NV_0.7_PF, △S=1.4406R), Na_3_Fe_0.5_Mn_0.5_V(PO_4_)_2_F_3_ (denoted as ME-NVPF, △S=1.02R).

***Synthesis of Na_3_Fe_0.1_Mn_0.2_Co_0.2_Ni_0.3_V_1.2_(PO_4_)_2_F_3_@CNTs***

Separation of Carbon Nanotubes: Multi-walled carbon nanotubes (CNTs) were ultrasonically dispersed in water at a stoichiometric ratio of 1:2 for 1 h. The dispersion was then transferred to a hydrothermal autoclave and reacted at 180 °C for 8 h. After the reaction solution cooled, it was subjected to filtration, and the collected solid was dried for 24 h to obtain dispersed CNTs.

Preparation of ME-NV_1.2_PF@CNTs: 0.025 g of CNTs was ultrasonically dispersed in 20 ml of water for 1 h. The dispersed solution was added to a mixed solution containing NH_4_VO_3_, ferrous acetate, manganese acetate, CoCl_4_·5H_2_O, nickel acetate, Na_2_H_2_PO_4_, and NaF. Lactic acid was used as the reducing agent, and C_8_H_18_O_5_ was used as the solvent. The mixture was stirred at 40 °C for 1 h and then transferred to a hydrothermal autoclave for a high-temperature reaction at 180 °C for 20 h. After the reaction, the solution was filtered, and the resulting solid was dried at 60 °C. The dried sample was ground uniformly, preheated at 200 °C for 4 h in a tube furnace, and then calcined at 400 °C for 6 h to obtain the final material.

**Materials Characterization**

The crystal structure of the samples was characterized using a powder X-ray diffractometer (XRD, Rigaku, Japan) with CuKα radiation (λ = 0.154 nm), yielding diffraction patterns of the series of medium-entropy materials. The microstructure and morphology of the materials were observed using field-emission scanning electron microscopy (SEM, JSM7500F) and transmission electron microscopy (TEM, JEM-2100). The particle size distribution was determined by laser diffraction using a Malvern Mastersizer series analyzer (Malvern Panalytical, UK). Energy-dispersive X-ray spectroscopy (EDS) images were recorded using an EDS system attached to the SEM instrument. X-ray photoelectron spectroscopy (XPS) measurements were performed using a PHI QUANTERA-II SXM instrument, combined with inductively coupled plasma (ICP) testing to analyze the elemental composition ratios and specific valence states of the metal elements. Thermogravimetric analysis (TGA, HQT-4) was conducted under a nitrogen atmosphere at a heating rate of 10 °C/min to analyze the thermal decomposition behavior of the samples, with a temperature range of 0-800 °C. The characteristic chemical structures of the sample molecules were analyzed using a Raman spectrometer (Renishaw In Via, UK).

**Electrochemical Measurement**

The electrochemical performance of the series of medium-entropy NVPF materials was evaluated by assembling 2032 coin cells in a glovebox with both water and oxygen content below 0.1 ppm. The working electrode was composed of active material, conductive agents (acetylene black and graphene), and binders (polyvinylidene fluoride (PVDF) and polyacrylic acid (TAA)) in weight ratios of 7:2 (3:1):1 (2:3). The uniformly mixed electrode slurry was coated onto aluminum foil and dried under vacuum at 60 °C for 8 h. The resulting aluminum foil film was then pressed into circular electrodes with a diameter of 12 mm, ensuring a mass loading of 1-1.5 mg/cm² for each electrode.

The coin cells were assembled using sodium metal as the counter electrode, glass fiber (Whatman GF/A) as the separator, and 1 M NaClO4 in DEC:EC = 1:1 vol% with 5% fluoroethylene carbonate (FEC) as the electrolyte. The charge-discharge curves, rate performance, cycling performance, direct current (DC) impedance, and galvanostatic intermittent titration technique (GITT) curves of the assembled cells were tested using a LAND battery testing system (CT2001A, Wuhan, China) within a voltage window of 1.5-4.3 V. Cyclic voltammetry (CV) curves and electrochemical impedance spectroscopy (EIS) curves were measured using a CHI-760E electrochemical workstation.

**Computational Methods**

We have employed the first-principles ^[1, 2]^ to perform all density functional theory (DFT) calculations within the generalized gradient approximation (GGA) using the Perdew-Burke-Ernzerhof (PBE)^[3]^ formulation. We have chosen the projected augmented wave (PAW) potentials^[4, 5]^ to describe the ionic cores and take valence electrons into account using a plane wave basis set with a kinetic energy cutoff of 520 eV. Partial occupancies of the Kohn−Sham orbitals were allowed using the Gaussian smearing method with a width of 0.05 eV. The electronic energy was considered self-consistent when the energy change was smaller than 10^−6^ eV. A geometry optimization was considered convergent when the energy change was smaller than 0.03 eV Å^−1^. During structural optimizations, a 2×2×1 k-point grid in the Brillouin zone was used for k-point sampling, and all atoms were allowed to relax. In our structure, the U correction is used for V (5 eV) atoms. Na spillover pathway can be calculated using the nudged elastic band (NEB) method with the transition state of an elementary reaction step. In the NEB method, the path between the reactant(s) and product(s) was discretized into a series structural images. The intermediate images were relaxed until the perpendicular forces were smaller than 0.05 eV/Å.


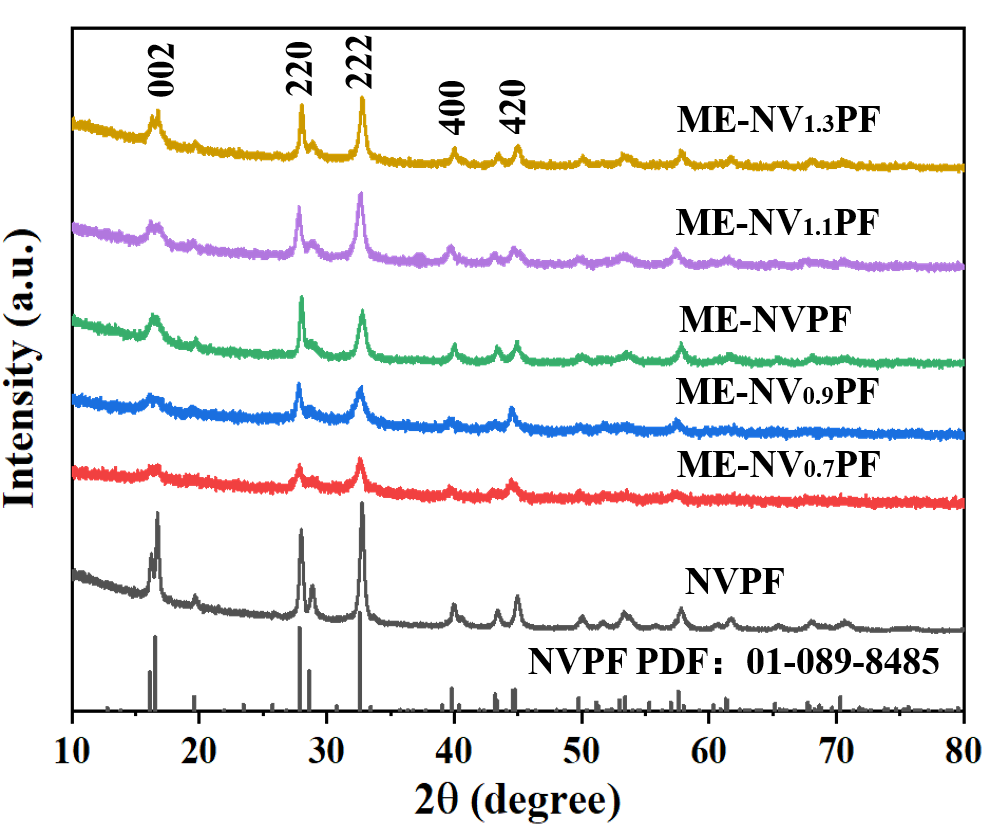


**Figure S1** The XRD spectra of NVPF and five medium-entropy samples.


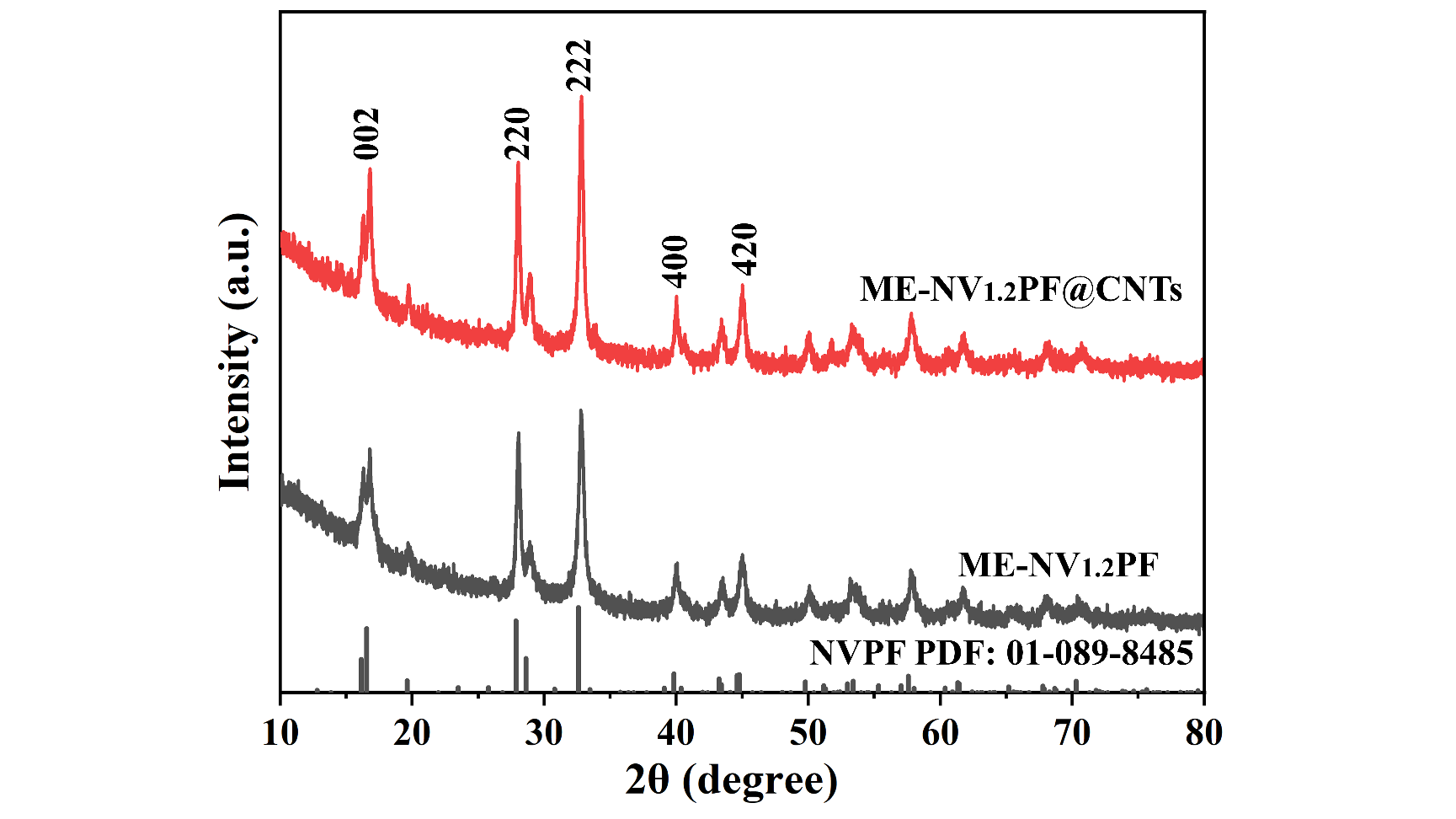


**Figure S2** The XRD spectra of ME-NV1.2PF and ME-NV1.2PF@CNTs samples.

**Table S1** The obtained crystallographic data of the ME-NV_1.2_PF sample.

space group: *P42/mnm*

*a* = *b* =9.03580 Å, *c* = 10.62530 Å, V= 867.509891 Å^3^

Weighted profile R-factor, R_p_ = 2.34%, Rwp = 2.91%.

| Atom | Wyckoff site | X | Y | Z | Occupancy |
| --- | --- | --- | --- | --- | --- |
| F2 | 8j | 0.26490 | 0.26490 | 0.37020 | 1.00 |
| F1 | 4f | 0.23170 | 0.23170 | 0.00000 | 1.08 |
| O2 | 8j | 0.07460 | 0.07460 | 0.15350 | 1.00 |
| Na2 | 8i | 0.79570 | 0.01260 | 0.00000 | 0.59 |
| P1 | 4d | 0.00000 | 0.50000 | 0.25000 | 1.05 |
| O3 | 8j | 0.39010 | 0.39010 | 0.16590 | 1.00 |
| P2 | 4e | 0.00000 | 0.00000 | 0.24450 | 1.00 |
| O1 | 16k | 0.10620 | 0.40100 | 0.16920 | 1.00 |
| Na1 | 8i | 0.53150 | 0.25080 | 0.00000 | 1.11 |
| V1 | 8j | 0.23750 | 0.23750 | 0.19670 | 0.60 |
| Fe1 | 8j | 0.23750 | 0.23750 | 0.19670 | 0.05 |
| Mn1 | 8j | 0.23750 | 0.23750 | 0.19670 | 0.10 |
| Co1 | 8j | 0.23750 | 0.23750 | 0.19670 | 0.10 |
| Ni1 | 8j | 0.23750 | 0.23750 | 0.19670 | 0.15 |

**Table S2** The obtained crystallographic data of the standard NVPF sample.

space group: *P42/mnm*

*a* = *b* =9.047 Å, *c* = 10.705 Å, V= 876.185 Å^3^

| Atom | Wyckoff site | X | Y | Z | Occupancy |
| --- | --- | --- | --- | --- | --- |
| F2 | 8j | 0.24660 | 0.24660 | 0.36420 | 1.00 |
| F1 | 4f | 0.24760 | 0.24760 | 0.00000 | 1.00 |
| O2 | 8j | 0.09470 | 0.09470 | 0.16820 | 1.00 |
| Na2 | 8i | 0.80300 | 0.05120 | 0.00000 | 0.50 |
| P1 | 4d | 0.00000 | 0.50000 | 0.25000 | 1.00 |
| O3 | 8j | 0.40310 | 0.40310 | 0.16050 | 1.00 |
| P2 | 4e | 0.00000 | 0.00000 | 0.25530 | 1.00 |
| O1 | 16k | 0.09690 | 0.40590 | 0.16290 | 1.00 |
| Na1 | 8i | 0.52340 | 0.22990 | 0.00000 | 1.00 |
| V1 | 8j | 0.24783 | 0.24783 | 0.18845 | 0.60 |

**Table S3** ICP test results of each sample material

| Sample | Theoretical content (%) | | | | | Actual content (%) | | | | |
| --- | --- | --- | --- | --- | --- | --- | --- | --- | --- | --- |
|  | Fe | Mn | Co | Ni | V | Fe | Mn | Co | Ni | V |
| NVPF | 0 | 0 | 0 | 0 | 24.40 | 0 | 0 | 0 | 0 | 24.32 |
| ME-NVPF | 6.63 | 6.51 | 0 | 0 | 12.07 | 6.67 | 6.52 | 0 | 0 | 12.32 |
| ME-NV_0.7_PF | 1.31 | 1.29 | 6.89 | 6.87 | 8.36 | 1.39 | 1.56 | 6.87 | 6.88 | 8.56 |
| ME-NV_0.9_PF | 1.32 | 2.58 | 5.54 | 5.52 | 10.79 | 1.39 | 2.48 | 5.83 | 5.79 | 10.58 |
| ME-NV_1.1_PF | 1.32 | 2.59 | 4.17 | 4.15 | 13.23 | 1.38 | 2.52 | 4.36 | 4.31 | 13.44 |
| ME-NV_1.3_PF | 1.32 | 2.60 | 2.78 | 4.16 | 14.46 | 1.39 | 2.62 | 2.83 | 4.12 | 14.36 |
| ME-NV_1.2_PF | 1.33 | 2.60 | 2.79 | 2.78 | 15.69 | 1.37 | 2.54 | 2.81 | 2.76 | 15.67 |
| ME-NV_1.2_PF@CNTs | 1.32 | 2.60 | 2.78 | 4.16 | 14.46 | 1.39 | 2.90 | 2.83 | 4.25 | 14.42 |


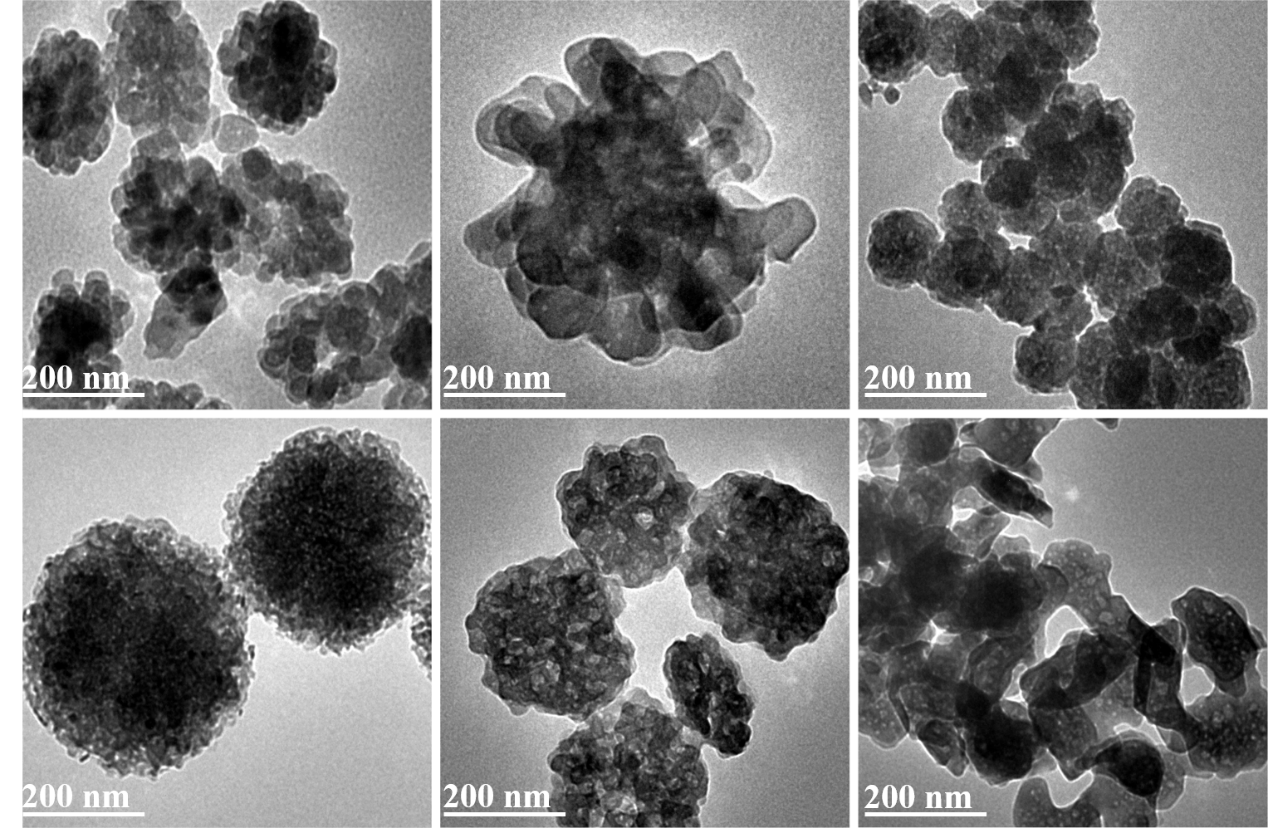


**Figure S3** TEM of (a) NVPF, (b) ME-NVPF, (c) ME-NV_0.7_PF, (d) ME-NV_0.9_PF, (e) ME-NV_1.1_PF and (f) ME-NV_1.3_PF.


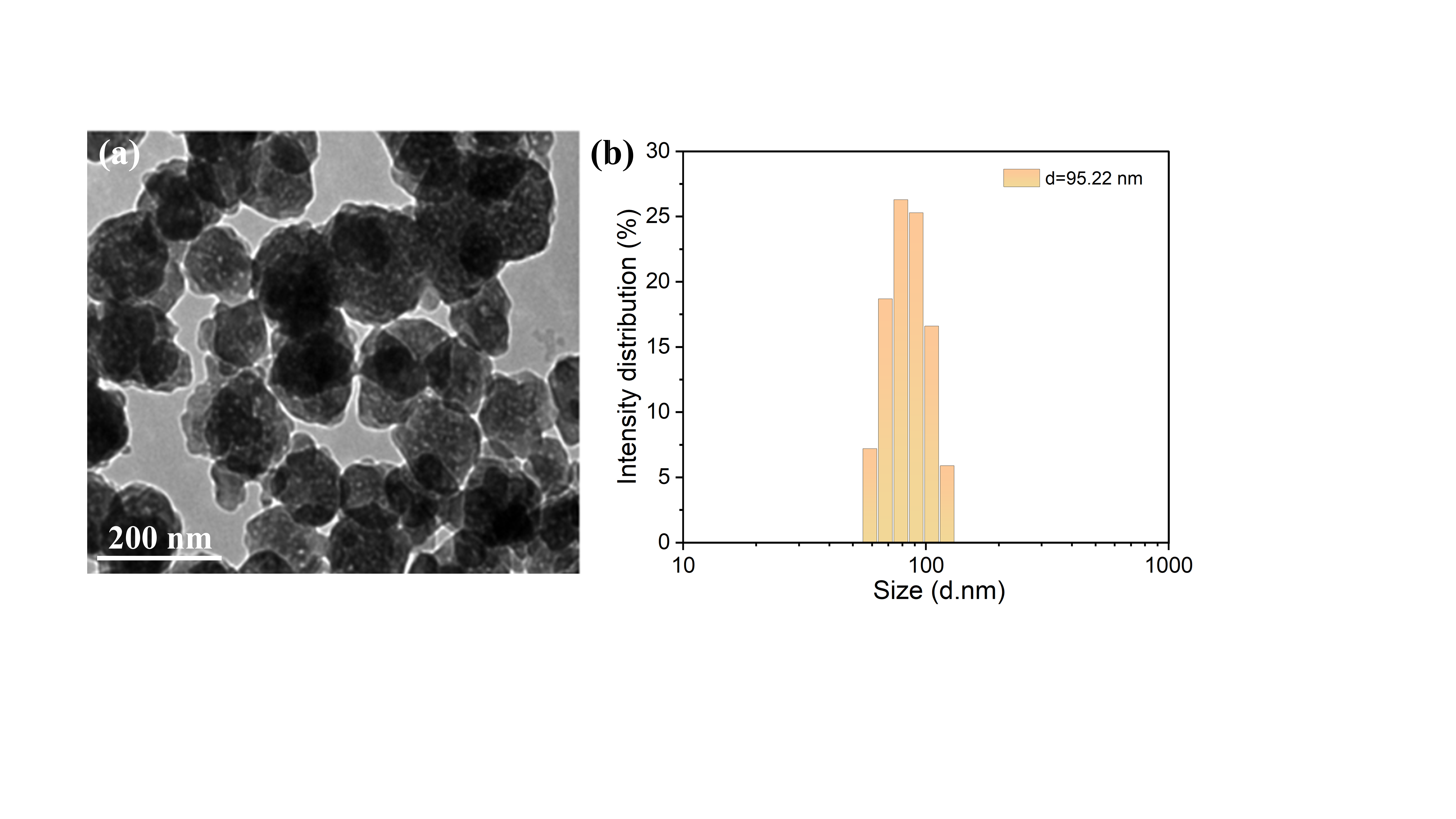


**Figure S4** (a) TEM of ME-NV_1.2_PF and (b) Size distribution of ME-NV_1.2_PF@CNTs.


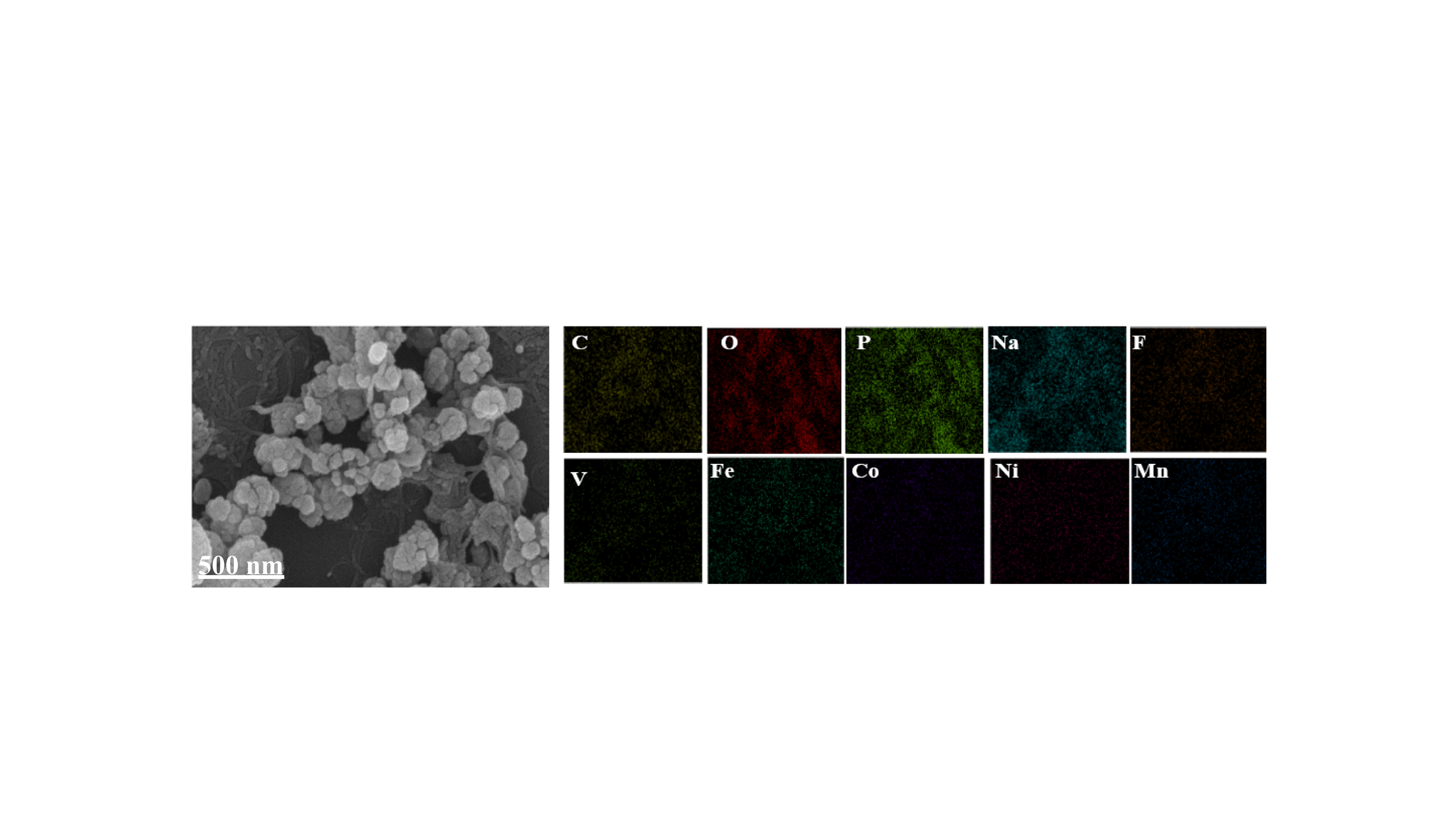


**Figure S5** EDS analysis of ME-NV_1.2_PF@CNTs.


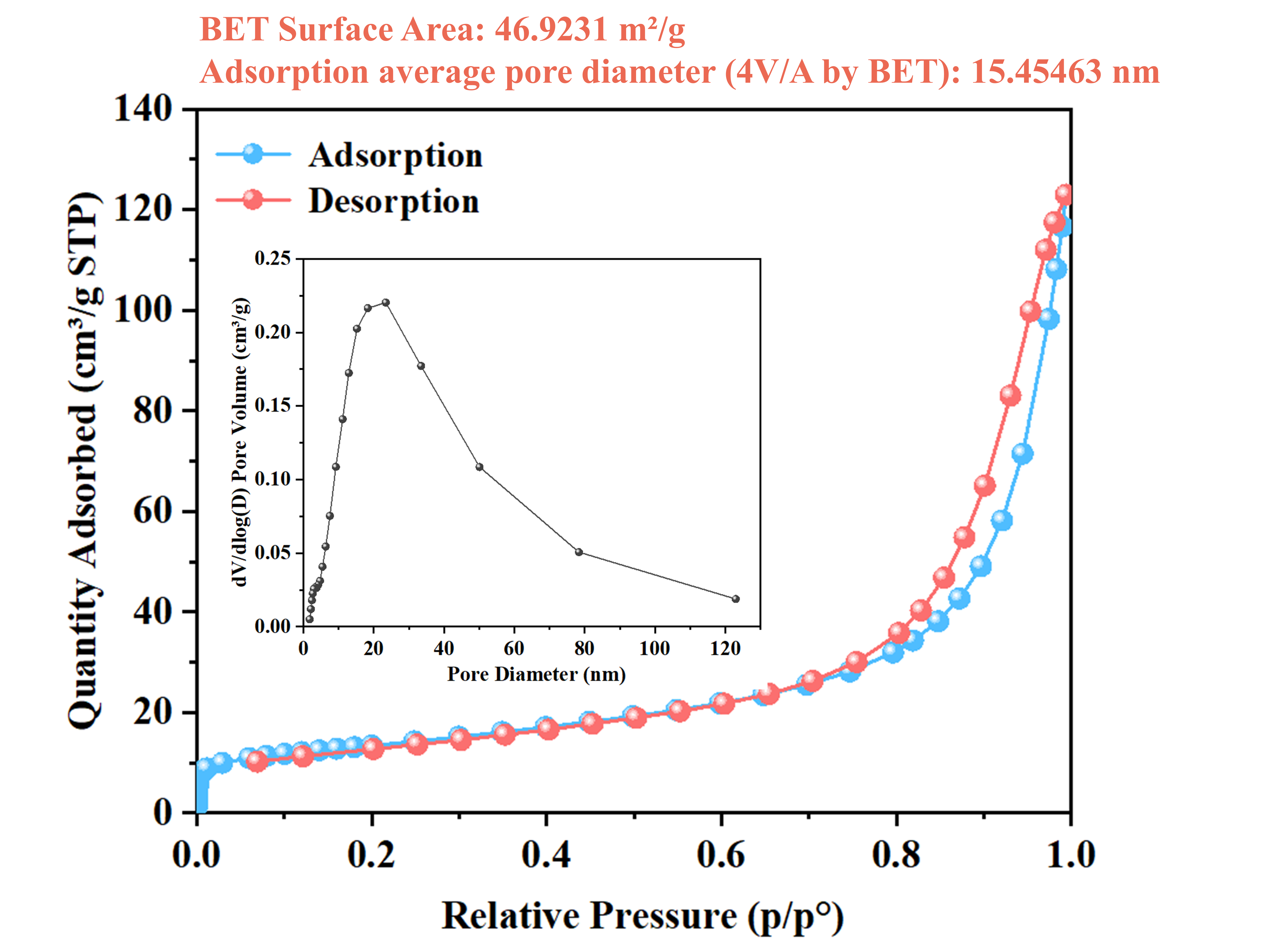


**Figure S6** BET test analysis diagram of ME-NV_1.2_PF@CNTs.


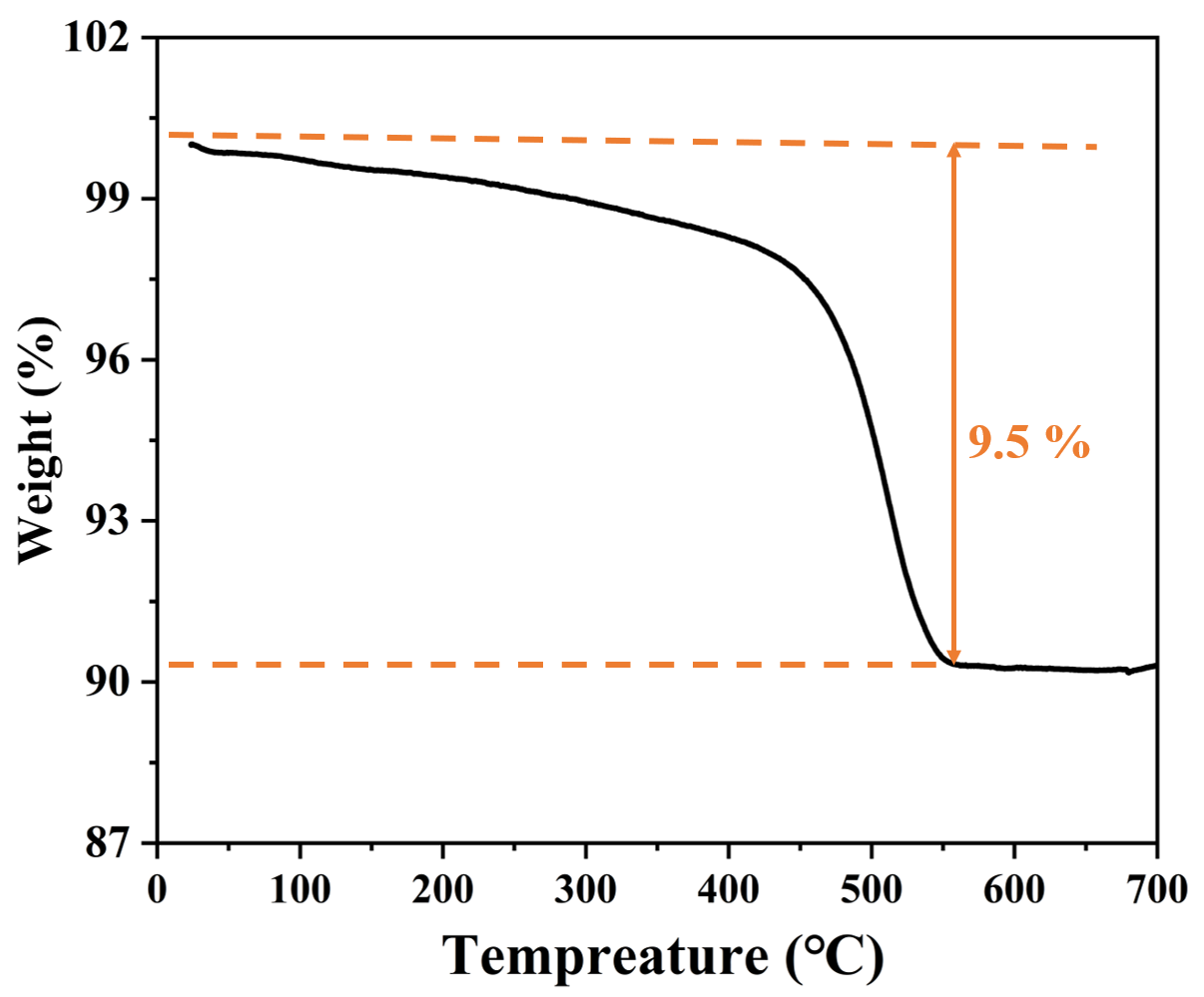


**Figure S7** TGA of ME-NV_1.2_PF@CNTs.


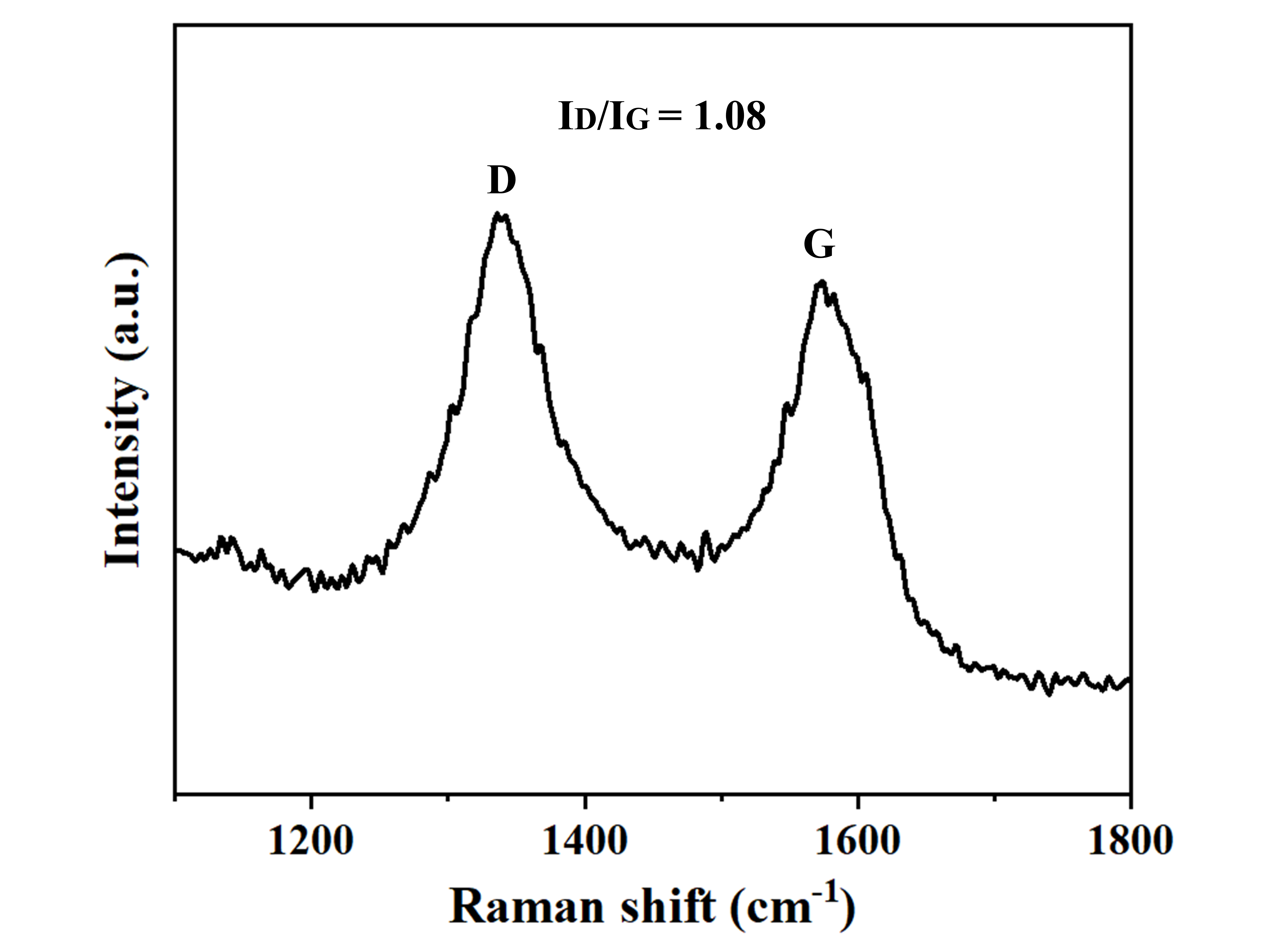


**Figure S8** Raman spectra of ME-NV_1.2_PF@CNTs


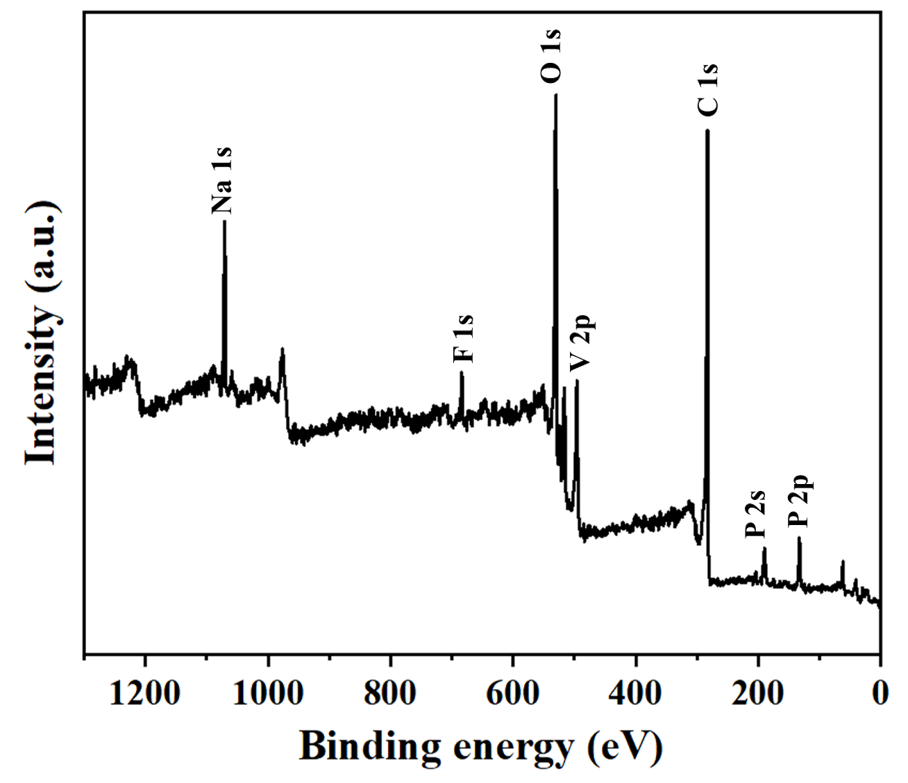


**Figure S9** XPS spectra of ME-NV_1.2_PF@CNTs


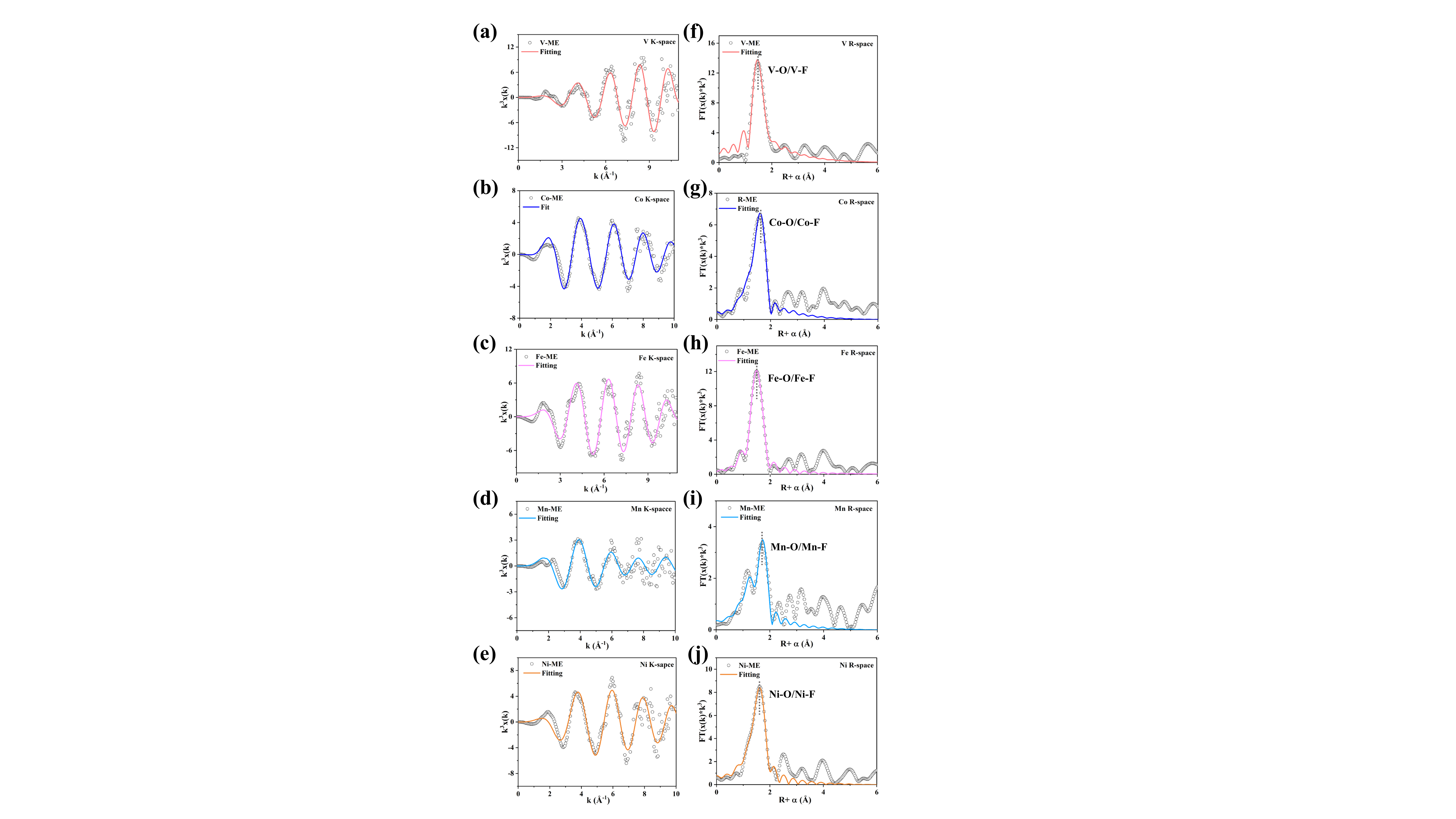


**Figure S10** (a-e) K-space and (f-j) R-space fitting profiles of five metals in ME-NV_1.2_PF@CNTs.

**Table S4** ^a^Curve fit parameters of K-edge EXAFS for ME-NV_1.2_PF@CNTs

| Path1 | ^b^R/Å | Path2 | ^b^R/Å | ^c^σ^2^/10^-3^Å^2^ | R-factor |
| --- | --- | --- | --- | --- | --- |
| Co-F | 2.095$\pm$0.04 | Co-O | 2.092$\pm$0.03 | 9.06$\pm$4.54 | 0.006 |
| Fe-F | 1.879$\pm$0.04 | Fe-O | 2.029$\pm$0.04 | 4.07$\pm$5.64 | 0.007 |
| Mn-F | 1.873$\pm$0.06 | Mn-O | 2.106$\pm$0.04 | 8.07$\pm$5.15 | 0.017 |
| Ni-F | 1.911$\pm$0.06 | Ni-O | 2.060$\pm$0.04 | 1.98$\pm$5.65 | 0.005 |
| V-F | 1.892$\pm$0.02 | V-O | 2.045$\pm$0.02 | 11.92$\pm$2.35 | 0.017 |

*^a^*The FT of EXAFS was derived with Athena program and the fitting of FTs was performed using Artemis software. S_0_ was fixed as 1.0. Data ranges: 2.0 < k < 10 Å^-1^, 1.0 < R < 2.0 Å^-1^. The number of variable parameters is 6.

*^b^R* is the distance between absorber and backscatter atoms.

*^c^σ*^2^ is the Debye-Waller factor. R-factor is residual factor.

**Table S5** Calculation results of diffusion resistance (R_ct_), warburg efficiency (σ) and sodium ion diffusion coefficient (D_Na+_) of ME-NV_1.2_PF@CNTs after 200, 400, 600 cycles.

| sample | R_ct_（Ω） | σ | D_Na_^+^(×10^-14^ cm^2^ S^-1^) |
| --- | --- | --- | --- |
| ME-V1.2PF@CNTs (200) | 495.2 | 117.38 | 20.5 |
| ME-V1.2PF@CNTs (400) | 748.5 | 161.95 | 10.7 |
| ME-V1.2PF@CNTs (600) | 890.7 | 201.25 | 6.95 |


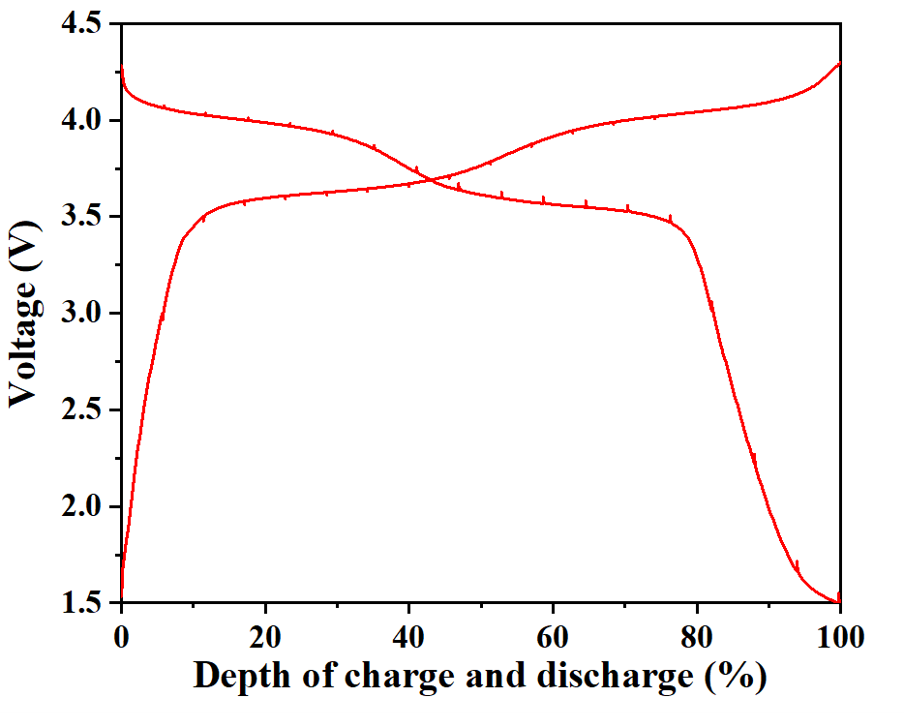


**Figure S11** The DC impedance test curve of ME-NV_1.2_PF@CNTs


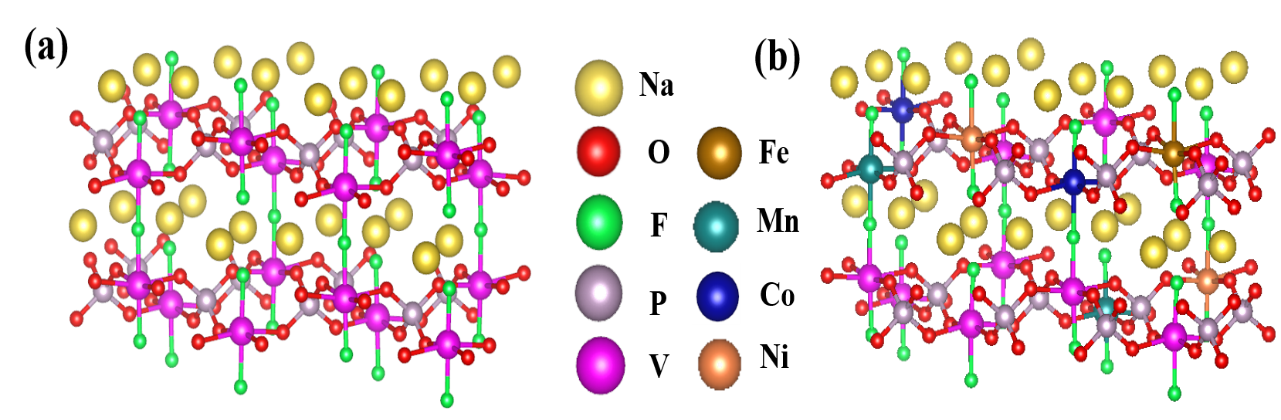


**Figure S12** The theoretical crystal models of (a) NVPF, (b) ME-NV_1.2_PF samples


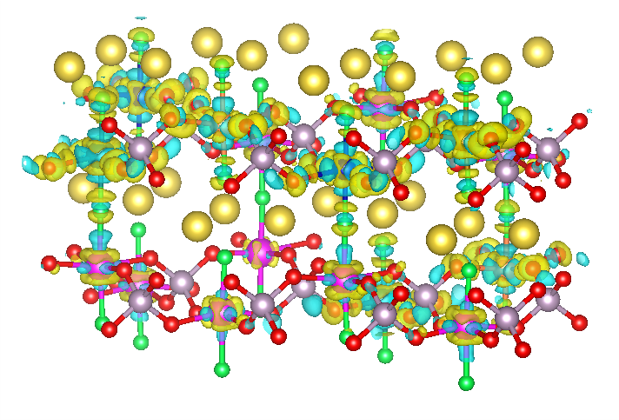


**Figure S13** The differential charge density diagram of ME-NV_1.2_PF sample (the isosurface value is 0.002 e/Å^3^. yellow is the accumulation area of electrons and blue is the dissipation area of electrons.


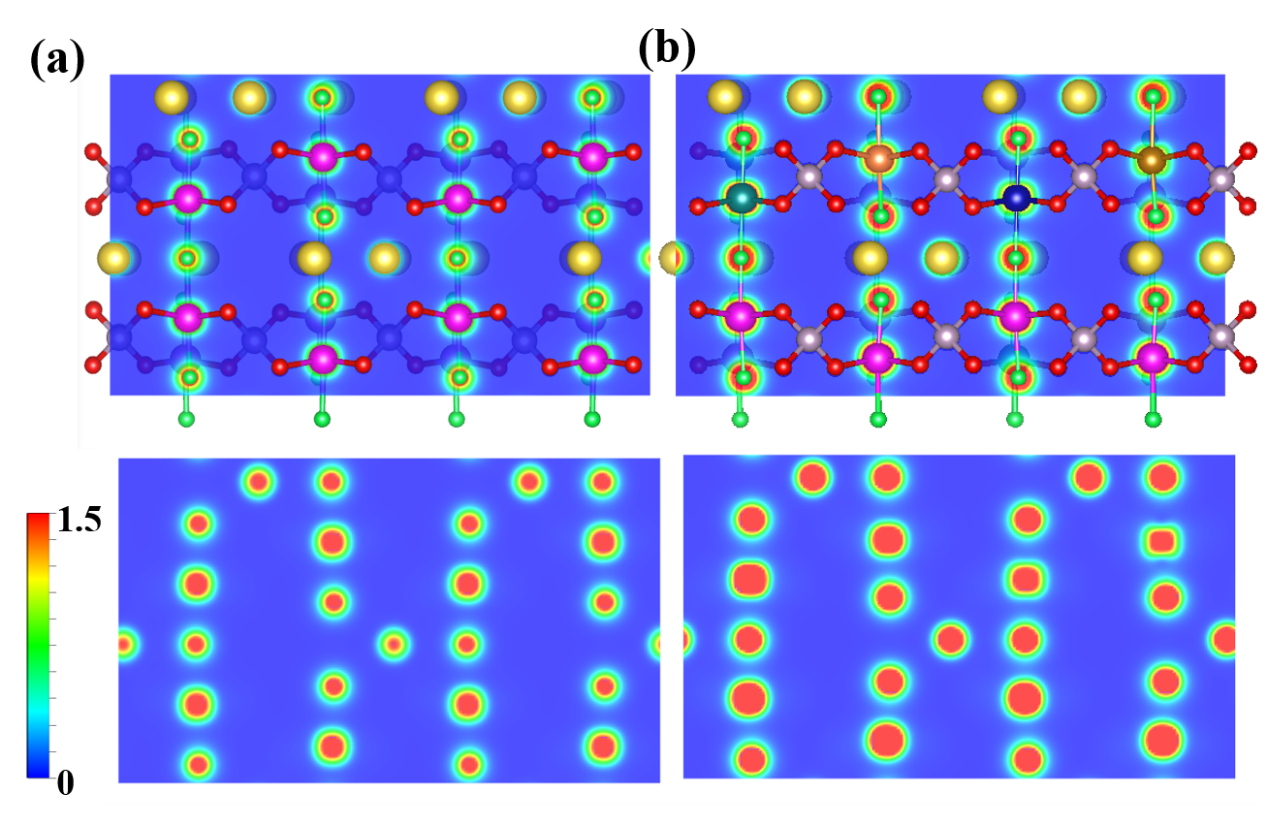


**Figure S14** The charge density distribution of (a) NVPF and (b) ME-NV_1.2_PF.


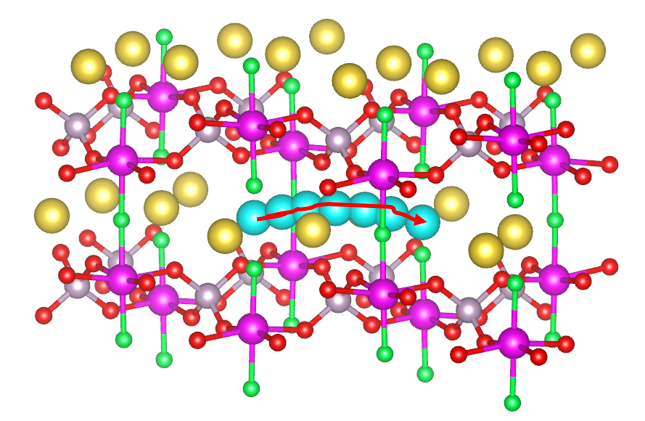


**Figure S15** The Na⁺ diffusion pathways within the NVPF structures

**Reference**

[1] G Kresse, J Furthmüller, *Comput. Mater. Sci.* **1996**, *6*, 15−50.

[2] G Kresse, J Furthmüller, *Phys. Rev. B* **1996**, *54*, 11169−11186.

[3] J P Perdew, K Burke, M Ernzerhof, *Phys. Rev. Lett.* **1996**, *77*, 3865−3868.

[4] G Kresse, D Joubert, *Phys. Rev. B* **1999**, *59*, 1758-1775.

[5] P E Blöchl, *Phys. Rev. B* **1994**, *50*, 17953−17979.
